# Whole genome sequencing of extreme phenotypes identifies variants in *CD101* and *UBE2V1* associated with increased risk of sexually acquired HIV-1

**DOI:** 10.1371/journal.ppat.1006703

**Published:** 2017-11-06

**Authors:** Romel D. Mackelprang, Michael J. Bamshad, Jessica X. Chong, Xuanlin Hou, Kati J. Buckingham, Kathryn Shively, Guy deBruyn, Nelly R. Mugo, James I. Mullins, M. Juliana McElrath, Jared M. Baeten, Connie Celum, Mary J. Emond, Jairam R. Lingappa, for the Partners in Prevention HSV/HIV Transmission Study and the Partners PrEP Study Teams

**Affiliations:** 1 Department of Global Health, University of Washington, Seattle, United States of America; 2 Department of Pediatrics, University of Washington, Seattle, United States of America; 3 Department of Genome Sciences, University of Washington, Seattle, United States of America; 4 Perinatal HIV Research Unit, University of Witwatersrand, Johannesburg, South Africa; 5 Partners in Health Research and Development, Kenya Medical Research Institute, Thika, Kenya; 6 Department of Microbiology, University of Washington, Seattle, United States of America; 7 Department of Medicine, University of Washington, Seattle, United States of America; 8 Vaccine and Infectious Disease Division, Fred Hutchinson Cancer Research Center, Seattle, United States of America; 9 Department of Epidemiology, University of Washington, Seattle, United States of America; 10 Department of Biostatistics, University of Washington, Seattle, United States of America; Vaccine Research Center, UNITED STATES

## Abstract

Host genetic variation modifying HIV-1 acquisition risk can inform development of HIV-1 prevention strategies. However, associations between rare or intermediate-frequency variants and HIV-1 acquisition are not well studied. We tested for the association between variation in genic regions and extreme HIV-1 acquisition phenotypes in 100 sub-Saharan Africans with whole genome sequencing data. Missense variants in immunoglobulin-like regions of *CD101* and, among women, one missense/5’ UTR variant in *UBE2V1*, were associated with increased HIV-1 acquisition risk (p = 1.9x10^-4^ and p = 3.7x10^-3^, respectively, for replication). Both of these genes are known to impact host inflammatory pathways. Effect sizes increased with exposure to HIV-1 after adjusting for the independent effect of increasing exposure on acquisition risk.

**Trial registration**: ClinicalTrials.gov NCT00194519; NCT00557245

## Author summary

Antiretroviral drugs for pre-exposure prophylaxis (PrEP) or treatment significantly reduce risk of HIV-1 acquisition and transmission, but face challenges of increasing access, maintaining high adherence, and selecting viral resistance. Improved understanding of the molecular determinants of HIV-1 sexual transmission could provide new public health HIV-1 prevention interventions. Factors proven to impact sexual HIV-1 transmission risk include epidemiologic exposure (e.g., level of virus in the transmitting partner and frequency of unprotected sex), presence of genital inflammation, and host genetic variants common in the population. Rare or intermediate frequency genetic variants are an increasingly recognized reservoir of complex human disease-causing factors, but are not well studied in HIV-1 infection. However, the low frequency of these variants reduces statistical power to detect disease associations. Aggregating variants in a common biological domain (e.g., a gene) can increase power for identifying variants with a common direction of effect. We report comparison of whole genome sequences from HIV-1 exposure extremes—highly-HIV-exposed individuals who remained HIV-uninfected and lower-exposed individuals who became HIV-infected. We discover and replicate associations between HIV-1 risk and aggregate variation in two genes, *CD101* and *UBE2V1* that increase directly with the level of HIV-1 exposure. These genes may modulate host inflammation thereby identifying molecular mechanisms linking genital inflammation to HIV-1 infection, possibly leading to novel candidate host-directed HIV-1 prevention interventions.

## Introduction

The discovery of the protective deletion variant, *CCR5*-delta32, in the chemokine receptor 5 gene, encoding a HIV-1 co-receptor [1–3], generated great enthusiasm to search for additional host genetic variants and pathways associated with HIV-1 acquisition as a means of identifying targets for new HIV-1 prevention and treatment strategies. This enthusiasm was further enhanced by documentation of HIV-1 exposed seronegative (HESN) individuals who had very high exposure and lacked *CCR5*-delta32, [4–7] suggesting existence of additional genetic factors that alter the risk of sexually transmitted HIV-1 infection (Online Mendelian Inheritance in Man [OMIM] phenotype #609423) [8]. At least one *in vitro* experiment supports the hypothesis of a strong genetic component to infection risk, reporting 50% heritability in cellular susceptibility to HIV-1 infection [9]. Nevertheless, genome-wide association studies (GWAS) to date searching for such genetic risk factors for HIV-1 infection risk have met with limited success [10–17].

Most GWAS have had moderate (~80%) average power to detect common variants, very low power to detect variants with minor allele frequency (MAF) = 5% (approximately 1% power for an OR = 2) and even less power to detect associated rare variants (RVs) (MAF≤1%). Additionally, susceptibility to HIV-1 can only be assessed among individuals who are exposed to the virus. While some assessment of HIV-1 exposure was used for most HIV-1 GWAS [10–17], exposure measurement error and/or exposure misclassification, including that related to lack of information about the HIV-1 infected partners’ plasma HIV-1 RNA level(s), can result in lower statistical power than anticipated. Furthermore, out of necessity, some studies have been forced to attempt replication across different ancestral/racial groups [10]. However, risk variants might differ between such groups, thereby lessening the power for replication. Hence, major gaps in HIV-1 genetic association studies still exist, and focus on power to detect rare associated variants employing high accuracy in HIV-1 exposure measurements is warranted. The contribution of rare variants to risk of HIV-1 acquisition is of particular interest because effect size is generally inversely correlated with MAF when an association does exist [18] and large effects can be expected to translate to strong interventional impact (e.g., HMG-CoA reductase inhibitors and familial hypercholesterolemia [19]).

With these issues in mind, we undertook an association study of HIV-1 acquisition using whole genome sequencing (WGS) of extreme phenotypes sampled from two large clinical trials and one observational study of African HIV-1 serodiscordant couples (stable heterosexual couples with one partner HIV-1-infected and the other partner HIV-1-seronegative at enrollment) (n = 8,593 couples). These studies (S1 Table) had similar clinical follow-up, including quarterly risk assessments, PCR-verification of HIV-1 infection, reports of protected and unprotected sexual activity from both partners, measurement of the infected partner’s plasma HIV-1 RNA level and molecular confirmation of transmission linkage through viral sequencing [20–22].

## Results

The study was conducted in two stages, Discovery and Replication (S1 Fig). In the Discovery stage we compared variation in genic regions from WGS data for 50 of the most highly HIV-1 exposed seronegative individuals (controls) to that for 50 of the most extreme low-exposure seroconverting individuals (cases; see Materials and methods). In the Replication stage, we employed a prospective design to test for association between time to seroconversion and candidate regions/variants passed on from the Discovery stage. Compared to other HESN not selected for the Discovery sample, the controls had higher average HIV-1 exposure scores, with higher enrollment plasma HIV-1 RNA level in the infected partner (4.9 vs 4.2 log_10_ copies/mL), a higher proportion of visits with unprotected sex reported by either partner (0.5 vs. 0), a lower percentage of circumcised men (33% vs. 54%) and longer follow-up time (Table 1).

**Table 1:** Characteristics* of Discovery sample.

Characteristic	Discovery Sample		Not selected for Discovery Sample	
	Seroconverter (n = 50)	HIV-1 exposed seronegative (n = 50)	Seroconverter (n = 73)**	HIV-1 exposed seronegative (n = 3692)**
Female gender	26 (52%)	26 (52%)	37 (51%)	1254 (34%)
Age (years)	28.8 (23.8,36.4)	27.2 (22.6,34)	29.9 (25.3,37.8)	33.5 (28,40.5)
Male circumcision^†^	10 (42%)	8 (33%)	13 (36%)	1328 (54%)
East African	36 (72%)	36 (72%)	44 (60%)	2520 (68%)
Study cohort:				
Couples Observational Cohort	8 (16%)	2 (4%)	7 (10%)	467 (13%)
Partners in Prevention HSV/HIV Transmission Study	42 (84%)	48 (96%)	66 (90%)	3225 (87%)
Enrollment clinical parameters:				
Plasma HIV-1 RNA (log10 c/mL)	4.8 (4.3,5.1)	4.9 (4.5,5.2)	4.8 (4.4,5)	4.2 (3.6,4.7)
CD4 count, cells/mL	435 (302,580)	416 (345,556)	417 (332,580)	457 (340,631)
Ever unprotected sex	36 (72%)	50 (100%)	50 (68%)	1745 (47%)
Proportion of visits with unprotected sex reported	0.4 (0,0.7)	0.5 (0.1,0.7)	0.4 (0,0.5)	0 (0,0.2)
Monthly exposure score^††^	1.3 (0.8,1.9)	1.7 (1.3,2)	1.4 (0.8,1.9)	0.2 (-0.6,0.8)
Cumulative monthly exposure score^#^	13 (5.7,21.4)	32.8 (24.7,44.9)	6.7 (2.6,15.5)	2.7 (-8.6,13.9)
Number of follow-up months	11.2 (6.3,14.6)	22.8 (16.9,23.3)	4.6 (2.9,8.7)	17.4 (11.7,23.2)

* Numbers and percentages are provided for categorical variables. Medians and inter-quartile ranges (IQR) are provided for continuous variables.

** Participants enrolled in Partners in Prevention HSV/HIV Transmission and Couples Observational Studies who were not selected for inclusion in the Discovery stage. Seroconverters are restricted to those confirmed by plasma viral sequence to be linked between transmitting and seroconverting partners. No Partners PrEP Study participants were included in the Discovery sample.

^**†**^ N and % of men only

^**††**^ Exposure score is an exponential scale normalized to 0 with a unit change in score equating to a 2.72-fold proportional change in HIV-1 acquisition risk. [23]

^#^ Cumulative months exposure scores are the sum of monthly exposures across all study months and provide a measure of the amount of risk sustained before either the seroconversion event (for cases) or before being censored from observation among those who remained HIV-1 seronegative.

Based on these factors, the controls had a 4.5-fold higher risk of infection than an average HESN individual from these cohorts [23]. Furthermore, control participants had comparable or higher exposure characteristics, including monthly exposure score, as compared to cases. However, given the extended time these control partners were followed, the cumulative HIV-1 exposure score sustained by these individuals was 2.5-fold greater than that of the cases (cumulative exposure scores = 32.8 versus 13, respectively, p < 1x10^-10^ via t-test). A group with higher-than-expected risk that nevertheless remains seronegative will, on average, comprise individuals with risk modifiers that are not accounted for in the calculated expectation, including genetic factors when they exist. Conversely, case participants, who acquired HIV-1 despite low estimated risk, were more likely than other participants to have factors, including genetic variants that predispose to HIV-1 infection risk.

### Discovery results

The 100 Discovery stage genomes were sequenced by Complete Genomics Inc. (CGI) with high quality results (S2 Table). The RVT1 test (“rare variant test 1”) [24], a statistical test designed specifically for rare variant association studies, was used to test the difference between extremes in functional variant burden (defined as the total number of minor alleles (aggregated variant scores) by gene comparing cases and controls; see Materials and methods) for each of 18,354 genic regions, including 284,632 functional variants in the tests. The regions with the two lowest RVT1 p-values (S2A Fig) had an estimated 83% probability that at least one of these was a true positive, based on a False Discovery Rate (FDR) analysis [25]. QQ-plots of the RVT1 results showed good adherence to expected behavior and no evidence of confounding by major ancestry nor by spatially-isolated pockets of ancestry [26] (S2B and S2C Fig). These two regions are transcribed regions of *CD101* (NCBI Gene ID: 9398) and *UBE2V1* (NCBI Gene ID: 7335). For both of these genes, individuals with greater numbers of polymorphic functional sites had increased risk of HIV-1 acquisition: *CD101* odds ratio (OR) = 2.7 (per functional site with at least one minor allele), 95% CI = [1.6–4.8], p = 3.6x10^-5^, and *UBE2V1* OR = 3.7, 95% CI = [1.8–7.5], p = 4.7x10^-5^ (Table 2).

**Table 2:** Nominal significance levels and effect sizes from Discovery stage for *CD101* and *UBE2V1* aggregate variant scores. Discovery stage results (N = 100, with 50 HIV-1 seroconversion events).

Gene	Burden^#^		OR^†^	95% CI	P
	Controls	Cases			
***CD101***	54	93	2.7	(1.6, 4.8)	3.6x10^-5^
***UBE2V1***	10	32	3.7	(1.8, 7.5)	4.5x10^-5^

^**# “**^Burden” is defined as the total number of minor alleles (aggregated variant scores) by gene separately in controls and cases.

^**†**^ RVT1 test OR = odds ratio is the multiplicative increase in odds of seroconverting per *CD101* functional variant site having at least one minor allele for an individual in the Discovery sample. [24].

P-values and odds ratios (ORs) in the discovery stage are from the RVT1 test of observed functional variants aggregated into one score for each individual. We employed an extreme phenotypes design with 50 seroconverters and 50 HIV-1-seronegative, highly-exposed individuals (retrospective design).

Within these two genes in the Discovery stage, eight variants were novel at the time of identification, and all eight validated by Sanger sequencing (S3 Table).

### Replication plan based on Discovery results

**Fig 1:**
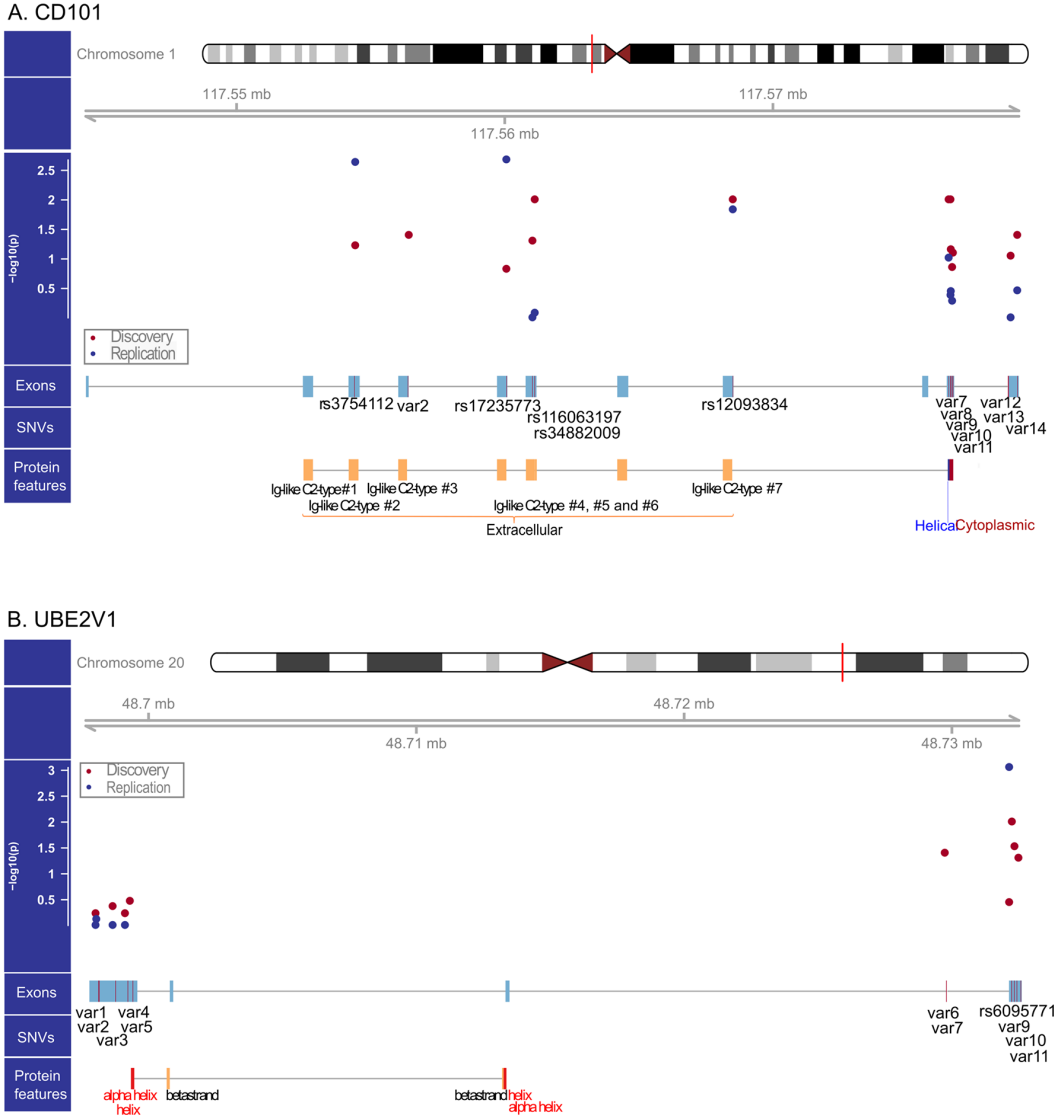
Genomic structure of *CD101* and *UBE2V1*, by-variant association significance, Primary Replication Variants, and protein features. Panel A: The Manhattan plot positioned below the chromosomal region map captures by-variant association significance for all single nucleotide variants (SNVs) present in *CD101* for both Discovery and Replication stage samples (red dots indicate the–log_10_(p) value for Discovery stage, and blue dots for Replication stage). rsIDs are shown below exon symbols only for the 5 Ig-like domain variants for *CD101* and the single 3’-UTR variant for *UBE2V1* that were associated with HIV-1 acquisition in the Replication analysis. The remaining Primary Replication Variants (PRVs) are indicated as “var”. The rsIDs for the *CD101* PRVs are: var2 = rs142460852, var7 = rs12097758, var8 = rs12067543, var9 = rs34248572, var10 = rs150494742, var11 = novel, var12 = rs2296448, var13 = novel, and var14 = rs35163967. Panel B: The Discovery and Replication stage SNVs for *UBE2V1*. rsIDs are shown below the single 3’-UTR variant (rs6095771) that was associated with HIV-1 acquisition risk in the Replication analysis. The remaining Primary Replication Variants (PRVs) are indicated as “var”. The rsIDs for the *UBE2V1* PRVs are: var1 = rs186621934, var2 = rs115164526, var3 = rs6095755, var4 = rs41283596, var5 = rs187204768, var6 = rs193169918, var7 = rs372425380, var9 = rs73278517, var10 = novel, var11 = rs185632114.

Based on the FDR results showing >80% chance of a true positive, the *CD101* and *UBE2V1* regions were moved forward to the Replication stage for testing in a longitudinal analysis. Test regions from the Discovery stage almost certainly include variants significantly associated, as well as, variants not significantly associated (i.e., noise) with outcome. The benefits, and perhaps even necessity, of using biological knowledge to increase signal-to-noise ratio for rare variant replication is well recognized [27, 28]. To increase the signal-to-noise ratio and increase power, we prioritized and grouped variants (see Materials and methods) in *CD101* and *UBE2V1* for Replication stage testing. Fourteen functional variants in *CD101* were designated as “primary replication variants” (PRVs) based on a direction of effect consistent with that for the Discovery result (S4 Table) and their degree of significance in by-variant tests (S3A Fig). These variants were subdivided into four sub-groups for replication testing (see Materials and methods): (1) five missense variants in regions encoding extracellular *CD101* immunoglobulin-like (Ig-like) protein domains [29], (2) five missense variants in the *CD101* cytoplasmic domain, (3) two 3’-UTR variants and (4) two splice site variants (Fig 1A, S3A Fig, S4 Table). Creating four separate *a priori* replication test groups increases the multiple-testing penalty, but is expected to further increase the signal-to-noise ratio within some of these variant test groups. Similarly, 11 of 15 predicted functional variants in *UBE2V1* were designated as PRVs, and these were divided into two groups: (1) six 5’-UTR and (2) five 3’-UTR variants (Fig 1B, S3B Fig, S5 Table). One of the 5’-UTR variants, rs6095771, is identified as a missense variant in the canonical transcript, and as a 5’-UTR for other transcripts. It was grouped with the 5’-UTR variants due to low predicted power to replicate a single variant with MAF = 0.02. Three of these variants were novel and another six are not found among Kenyans in the 1kG database [30] (S5 Table). Furthermore, two of the functional *UBE2V1* Discovery variants that are indexed in the ESP [31] or ExAC [32] databases are extremely rare and found in ESP only among individuals of African descent (rs187204768, MAF = 4/4046 and rs6095771, MAF = 20/4400) (S5 Table). These results indicate that the *UBE2V1* association could not be detected at the variant level using GWA-type methods and can only be detected in a population with substantial recent African ancestry, such as the present study.

**Fig 2:**
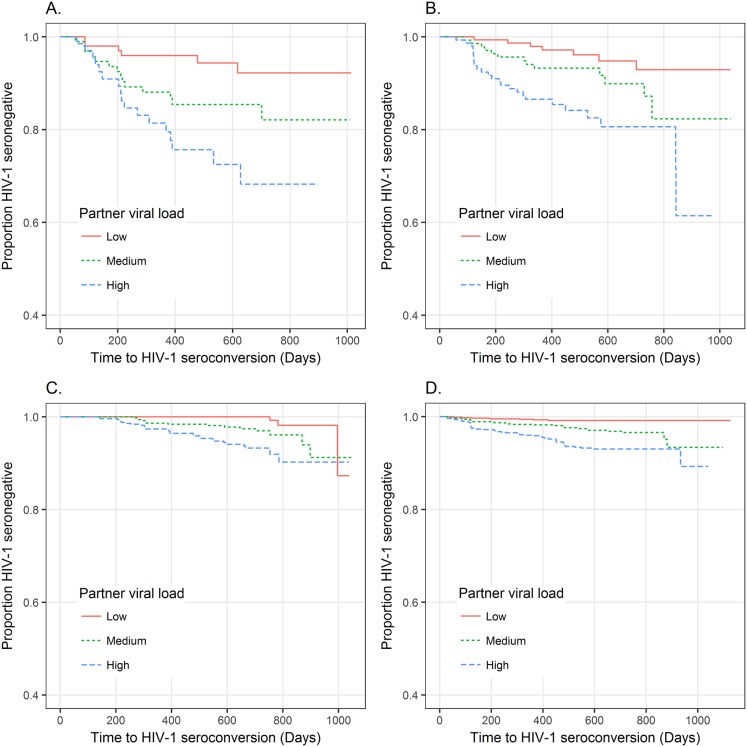
Time to seroconversion by Protected sex Index (PI) and tertiles of partner HIV-1 viral load distribution. The protected sex index (PI) is the proportion of visits at which the female partner reported always having protected sexual intercourse with the HIV-1 infected partner (= 1 –proportion of interviews with reported unprotected sex by the female partner.) The figures show time to seroconversion stratified by tertiles of plasma viral load of the HIV-1 infected partners in log_10_ copies/mL: Low = (0, 3.8], Medium = (3.8, 4.5], and High = (4.5,8]. Panels show this relationship for by PI strata: Panel A: PI = (0, 0.3], Panel B: PI = (0.3 to 0.6], Panel C: PI = (0.6 to 1.0), and Panel D: PI = 1.0. PI shows reasonable accuracy in predicting overall risk of seroconversion, indicating this variable can be used to eliminate individuals with probably little or no exposure from the study samples although a few more highly exposed individuals might also be dropped from the analysis.

Across these three HIV-1 serodiscordant couples cohorts, the pool of participants who were available for the Replication stage had varying amounts of reported sexual exposure. Because inclusion of individuals with no or little HIV-1 exposure could reduce power, we identified and excluded these individuals using a Protected-sex Index (PI). PI is defined as the proportion of study visits for which only abstinence or 100% condom use was reported; we considered this our measure of baseline behavior tendency. PI predicted overall HIV-1 seroconversion rates among the three HIV-1 cohorts (Fig 2), indicating reasonably low measurement error and a high signal-to-noise ratio for PI. Simulation studies predicted that power would be maximized when only individuals with PI ≤0.6 were included in the Replication analysis, despite a larger sample size when individuals with lower exposure are included (see Materials and methods). Accordingly, the Replication cohort was restricted to the 261 individuals HIV-1 uninfected at baseline, who each had PI ≤ 0.6 and were not selected for the Discovery stage analysis.

### Replication results

Genotyping of variants in *CD101* and *UBE2V1* for the 261 Replication stage individuals was completed using molecular inversion probe sequencing (MIPs) [33] technology (see Materials and methods). Given the cost efficiency of using MIPs, an auxiliary sample of 968 participants selected by more common methods rather than sexual exposure also was sequenced and used for verification of simulated power studies (see Materials and methods) for a total of 1229 individuals with MIPs data for *CD101* and *UBE2V1* (S6 Table). Neither *CD101* splice variant from the Discovery stage was found in the Replication sample (S7A Table), reducing the number of *CD101* primary test groups from four to three. In total, 83 *CD101* SNVs were detected among the 1229 individuals (S8 Table). For *UBE2V1*, three of five 3’-UTR PRVs were found in the Replication sample but only one of the six 5’-UTR PRVs was present (S7B & S9 Tables). This is consistent both with the rarity of these *UBE2V1* variants and enrichment for these variants in the Discovery sample.

#### *CD101* primary replication

**Fig 3:**
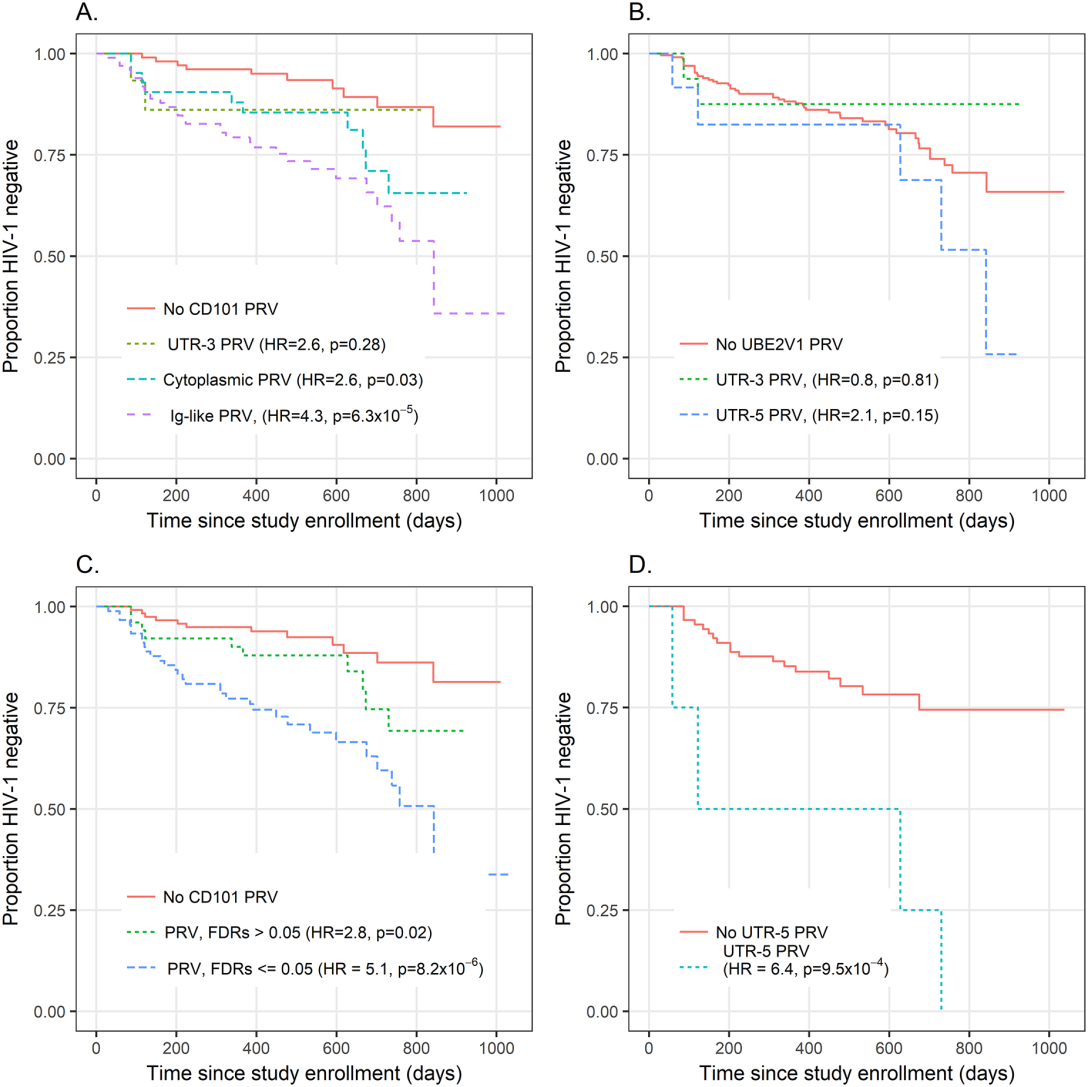
Results of Primary Replication Variant group tests for *CD101* and *UBE2V1*. Panel A: *CD101* Primary Replication Variant (PRV) groups—All three PRV groups present in the Replication stage are shown in the Kaplan-Meier plot. The *CD101* Ig-like PRV group is significantly associated with a higher rate of seroconversion after adjustment for multiple testing (p = 1.9 x 10^−4^), while the 3’-UTR and cytoplasmic PRV tests were not significant. Panel B:*UBE2V1* PRV groups—Four variants with False Discovery Rates (FDRs) < 0.05 in a by-variant analysis of Replication stage variants were grouped (red) for visual exploration of effect size. Panel C: *CD101* PRVs by FDR grouping–*CD101* variants grouped by observed FDR in Replication stage. Panel D: *UBE2V1* UTR-5’ PRV, Females—The hazard ratio for presence of the minor allele for rs6095771 in females is shown, which is significant after adjustment for four tests (two variant groups and two sexes, HR = 6.4, nominal p = 1.2x10^-3^, adjusted p = 4.8x10^-3^).

In the primary replication testing, association between the *CD101* Ig-like PRV group and the hazard for seroconversion was significant (HR = 4.3, p = 2.1x10^-4^ after Bonferroni correction) (Table 3; Fig 3A); the result was unchanged after adjustment for sex, age, cohort and country/ethnic group (8 self-identified groups; S4 Fig). Country/ethnic group serves as an adjustment variable for potential confounding by ancestry group, and no such evidence of confounding was apparent as the estimates were virtually unchanged by this adjustment (Table 3). Principal components were available on a subset of highly exposed Replication individuals, and applying the Cox model to this subset with three PCs in the model rather than country/ethnic group gave similar results (S4 Fig). There was no sex interaction or age interaction present for the *CD101* Ig-like PRV score. When bacterial vaginosis (BV) was tested as a confounder among a subset of 138 women in the Replication plus auxiliary (Augmented) sample it increased the effect size (HR = 3.4, p = 0.02 before adjustment; HR = 4.1, p = 0.01 with adjustment) and therefore we did not include it as a confounder. The *CD101* cytoplasmic PRV group was nominally significant (HR = 2.8, p = 0.03) (Table 3; Fig 3A), but not after Bonferroni adjustment for multiple testing (p = 0.09). No other *CD101* PRV group was found to be significant after adjustment for multiple testing (Table 3).

**Table 3:** Nominal significance levels and effect sizes from Replication stage for *CD101* and *UBE2V1* aggregate variant scores. Primary Replication Results (N = 261, with 53 HIV-1 seroconversion events).

Primary Replication Variant (PRV) group	n^†^	HR^††^	95% CI	P
*CD101* 3’-UTR only	15	2.3	(0.5, 10.7)	0.28
*CD101* Cytoplasmic (no Ig-like)	42	2.6	(1.1, 6.3)	0.03
*CD101* Ig-like	99	4.3^#^	(2.1, 8.9)	6.3x10^-5^ *
*UBE2V1* 3’-UTR	16	0.8	(0.2, 3.5)	0.81
*UBE2V1* 5’-UTR	10	2.1	(0.8, 5.9)	0.15
*UBE2V1* 3’-UTR, females only	10	1.0	(0.1, 7.6)	0.99
*UBE2V1* 5’-UTR, females only	4	6.4^##^	(2.1, 19.1)	9.5x10^-4^ **

^†^ n = number of the 261 individuals carrying a PRV in the indicated PRV group.

^**††**^ Cox model HR = hazard ratio is the multiplicative increase in “instantaneous” relative risk for seroconversion for an individual having at least one minor allele in the PRV group compared to individuals with no PRV

^#^ HR = 4.3 (nominal p = 6.96x10^-5^, Bonferroni multiple-test p = 2.1 x 10^−4^) after adjustment for cohort, sex, age and country-tribe.

^##^ HR = 5.14 (nominal p = 0.011, Bonferroni multiple-test p = 0.048) after adjustment for cohort, sex, age and country-tribe.

*P = 1.9x10^-4^ after Bonferroni correction for multiple testing.

** P = 3.7x10^-3^ after Bonferroni correction for multiple testing.

PRVs in the Replication sample in *CD101* are: 3’-UTR: 1_117578861, rs35163967; cytoplasmic: rs12097758, rs12067543, rs34248572, rs150494742; Ig-like: rs3754112, rs17235773, rs116063197, rs12093834, rs34882009. For *UBE2V1* Replication PRVs are: 3’-UTR: rs115164526, rs6095755, rs186621934, rs41283596; 5’-`UTR: rs6095771

P-values and hazard ratios (HRs) in the Replication stage are from the Cox model to test association between time-to-seroconversion and presence of a minor allele for a PRV. PRVs were selected by individual significance level from the Discovery stage and subseted by function or protein domain.

#### CD101 exploratory results

For comparison with the Replication Cox model results for *CD101*, we applied the Cox model to the same *CD101* Ig-like variant group in the Discovery stage sample and found a similar result: HR = 3.4 (95% CI = [1.8–6.1]; p = 7.3x10^-5^). While the primary replication was limited to variants found in the Discovery stage, we performed exploratory analyses on all *CD101* variants in the Replication stage and found that when assessed individually, three of the Ig-like PRVs found in the Replication sample had FDRs < 0.05 (rs17235773, rs3754112, rs12093834; S5 Fig) with HRs of 3.1 (p = 0.002), 3.1 (p = 0.002) and 2.0 (p = 0.02), respectively (S7A & S9 Tables). Among the 13 *CD101* variants annotated by the Variant Effect Predictor (VEP) [34] as either missense or regulatory, and which were identified among individuals in the Replication Sample, the two most significant in this by-variant analysis (rs17235773 and rs3754112) pass a Bonferroni bound for significance (p < 0.0038), providing additional strong evidence for the association of *CD101* variants with HIV-1 acquisition. A fourth Ig-like missense variant found only in the Replication sample (rs140567712) also had an individual-variant level FDR < 0.05 with HR = 6.9 (p = 0.009) and CADD score [35] = 16.9 (Fig 2; S8 Table).

#### *UBE2V1* primary replication

Association with the *UBE2V1* 3’-UTR PRV group was not significant. However, among the six 5’-UTR variants present in the Discovery sample, the only one identified in the Replication sample (rs6095771) was significantly associated with increased risk in women after adjustment for the two PRV group tests and testing within sex groups (HR = 6.4, nominal p = 9.5x10^-4^, adjusted p = 3.7x10^-3^; Fig 3B & 3D; Table 3.) This also is the only *UBE2V1* Discovery variant that is missense in some transcripts; it has a very high CADD score and is predicted to have a moderate to deleterious impact on function (S5 Table). Because there are only 4 women with this variant in the Replication sample, the reliability of the Cox model p-value (an asymptotic, not exact p-value) is uncertain. Hence, we also tested the association between seroconversion and this variant using Fisher’s exact test. Given a total of 122 women in the Replication sample, 22 of whom seroconverted, the probability under the null hypothesis (of no association with seroconversion) that all 4 who carry the rare rs6095771 variant are found among the 22 seroconverters is only 0.001 (≤ 0.004 after correction for 4 tests), providing substantial evidence for a true association between this variant and higher risk of seroconversion in females. None of the six males carrying this variant in the Replication sample seroconverted.

### Dose-response and modeling of sexual exposure

To test for further evidence of a true positive association between HIV-1 acquisition risk in women and Ig-like *CD101* variants or *UBE2V1* rs6095771, we augmented the Replication data with variant data from the auxiliary sample and tested for an interaction between the reported PI score and each PRV score to ascertain whether a dose-response relationship was present. “Dose” here is HIV-1 virus exposure quantified in terms of frequency of unprotected sex with a partner with the average plasma HIV-1 RNA in the Replication sample partners. For *UBE2V1* rs6095771, this increased the number of women with 5’-UTR PRVs in the analysis from 4 to 28 (S7B Table).

**Fig 4:**
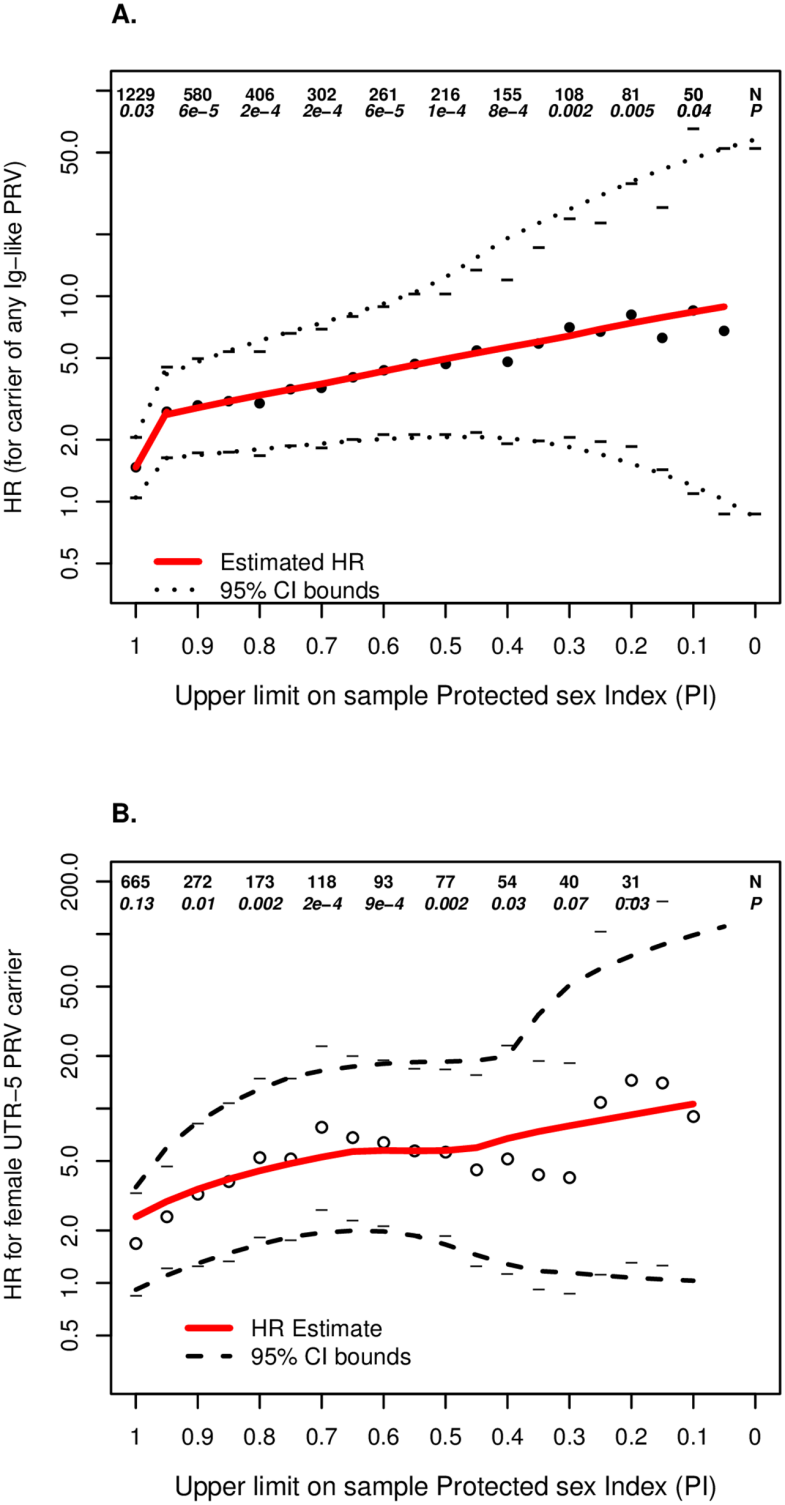
Estimated hazard ratio by Protective Index (PI) cut-off defining the analysis sample. Panel A: *CD101* Ig-like missense variant group hazard ratio (HR) estimate increases with increased sexual exposure among the individuals from the augmented sample; Panel B: *UBE2V1* 5’-UTR Primary Replication Variant HR estimate increases with sexual exposure among the individuals from the augment sample. Two variants contribute to this analysis; see S7 Table. Numbers at the top of each plot indicate total sample size (N) included at that level of the plot, and the p-value (P) for the reported HR.

We found strong dose-response relationships for both genes, indicating that the association between variant scores and HIV-1 risk is positively related to the frequency of unprotected sex: p = 5.0x10^-8^ for the *CD101* Ig-like dose-response and p = 8.2x10^-6^ for the *UBE2V1* rs6095771 dose-response, with exposure assessed through the PI score and modeled on a log-scale (Fig 4; S7 Fig). Because the model is adjusted for PI, these significant associations with increasing dose are in addition to the effect of decreasing PI. Overall model significance levels including the variant and dose-response variables were marked at p = 5.1x10^-12^ and p = 1.7x10^-12^, respectively, for *CD101* and *UBE2V1* in explaining variation in HIV-1 seroconversion risk (Tables 4 and 5).

**Table 4:** Dose-response models (protection index (PI) × genetic variant interaction models) using the augmented sample.

*CD101 Ig-like Primary Replication Variant Results*						
**Full (Interaction) Model**	**Group N**	**HR**	**95% CI**	**P**	**Model P**	**LR Stat**
Presence of Ig-like PRV	447	5.4*	(1.9, 15.2)	1.4x10^-3^	--	--
Log.PI	NA	0.69	(0.27, 1.8)	5.0x10^-8^	--	--
Ig-like x log.PI interaction	NA	0.21	(0.07, 0.6)		--	--
N = 1229 (179 events)					5.1x10^-12^	35.19
**Reduced Model**	**Group N**	**HR**	**95% CI**	**P**	**Model P**	**LR Stat**
Presence of Ig-like PRV	447	1.5	(1.0, 2.0)	0.03	--	--
N = 1229 (179 events)					0.07	5.48

PRV = Primary Replication Variant (see Table 3); PI = protection index; log.PI = log(10 x PI + 1)– 1 (see S7 Fig); HR = hazard ratio; CI = confidence interval; P = p-value, where the p-value for the interaction is for joint p-value for the two non-orthogonal terms (individual p-values are not available); LR Stat = likelihood ratio statistic for the model.

*HR applies to individuals with PI = 0; HRs at other PI values can be calculated from the HR for log.PI and the interaction term.

Assessment of dose-response, when possible, provides strong evidence of causal association from observational data. Dose in this setting is defined as sexual exposure to the HIV-1 virus and estimated by condom-use behavior: the protection index (PI) is defined as the propensity to use condoms, estimated as the proportion of visit surveys at which only protected sex (or abstinence) with the infected partner was reported. When the interaction is taken into account using individuals in the analysis who were probably not exposed, the model p-values are highly significant (p < 1x10^-11^) but not significant when the interaction is not included. The reduced models also show that despite the larger sample of 1229 individuals compared to the n = 261 in Table 3, the test cannot detect these associations without accounting for unexposed individuals. The hazard-ratio in the interaction model is the estimate for those who report never using protection (condoms) and have the average amount of sexual contact among those who report never using condoms.

**Table 5:** Dose-response models (protection index (PI) × genetic variant interaction models) using the augmented sample.

*UBE2V1 5’-UTR Primary Replication Variant Results*						
**Full (Interaction) Model**	**Group N**	**HR**	**95% CI**	**P**	**Model P**	**LR Stat**
Presence of 5’-UTR PRV	28	16.2*	(3.6,72.6)	2.8x10^-4^	--	--
Log PI	NA	0.3	(0.1,0.6)	8.2x10^-6^	--	--
5’-UTR x log.PI interaction	NA	0.04	(0.004,0.4)		--	--
N = 1229 (179 events)					1.7x10^-12^	24.47
**Reduced Model**	**Group N**	**HR**	**95% CI**	**P**	**Model P**	**LR Stat**
Presence of 5’-UTR PRV	28	1.6	(0.7, 3.6)	0.28	--	--
N = 1229 (179 events)					0.27	1.04

PRV = Primary Replication Variant (see Table 3); PI = protection index; log.PI = log(10 x PI + 1)– 1 (see S7 Fig); HR = hazard ratio; CI = confidence interval; P = p-value, where the p-value for the interaction is for joint p-value for the two non-orthogonal terms (individual p-values are not available); LR Stat = likelihood ratio statistic for the model.

*HR applies to individuals with PI = 0; HRs at other PI values can be calculated from the HR for log.PI and the interaction term.

Assessment of dose-response, when possible, provides strong evidence of causal association from observational data. Dose in this setting is defined as sexual exposure to the HIV-1 virus and estimated by condom-use behavior: the protection index (PI) is defined as the propensity to use condoms, estimated as the proportion of visit surveys at which only protected sex (or abstinence) with the infected partner was reported. When the interaction is taken into account using individuals in the analysis who were probably not exposed, the model p-values are highly significant (p < 1x10^-11^) but not significant when the interaction is not included. The reduced models also show that despite the larger sample of 1229 individuals compared to the n = 261 in Table 3, the test cannot detect these associations without accounting for unexposed individuals. The hazard-ratio in the interaction model is the estimate for those who report never using protection (condoms) and have the average amount of sexual contact among those who report never using condoms.

The interaction models also provide estimated HRs for the risk groups under the assumptions of no protection and the average frequency of heterosexual intercourse among those in the Replication Stage (*CD101* HR = 5.4, p = 1.4x10^-3^, 95% CI = [1.9, 15.2]; and *UBE2V1* HR = 16.2, p = 2.8x10^-4^, 95% CI = [3.6, 72.6]) for the Ig-like PRV score and *UBE2V1* rs6095771, respectively. Again, these HRs are adjusted for PI, which means that the increase in effect size is above that explained by the PI variable.

### Power and effect size results when using N = 1229 individuals not accounting for sexual exposure

When we included all 1229 samples genotyped after the Discovery stage (Replication sample and auxiliary sample) in a model that does not account for sexual exposure/PI we found highly attenuated HRs (Tables 4 and 5) and non-significant p-values. This phenomenon occurs because the estimated HRs are averages over a group that contains individuals who have no risk due to no sexual exposure, “diluting” the effect we detected in those with higher exposure.

To illustrate these effects as a function of the exposure to HIV-1 viral quantity, HRs were estimated with step-wise decreases in sample size, excluding exposed individuals at each step (Fig 4, S6 & S7 Figs).

### Association between replicated CD101 Ig-like variants and plasma cytokine levels, and with plasma HIV-1 RNA set point

Levels of cytokines in blood are a useful measure for immunologic function and may indicate presence of a generalized host pro-inflammatory state [36]. We evaluated whether the three *CD101* Ig-like PRVs with FDR < 0.05 in the replication stage were also associated with altered plasma cytokine levels compared to individuals without any of these variants (see Materials and methods). Among the 163 individuals in this subset for whom *CD101* genotypes were determined and for whom measurements of 25 plasma cytokines were available [37], carriers of these *CD101* risk alleles had significantly lower serum levels of IL1R1 (right-shifted distribution) compared to those without any *CD101* risk alleles (OR = 0.19 for achieving the 75th percentile IL1R1 value, 95% CI = [0.07, 0.54], p = 1.7x10^-3^; adjusted p = 0.04) (S8 and S9 Figs). There was also a tendency toward lower levels of sCD40L (p = 0.0049; S10 Table). Frequencies of the *UBE2V1* 5’-UTR PRVs were too low in the individuals with cytokine measurements to allow for assessment of any association with cytokine levels.

None of these variants were associated with HIV-1 plasma RNA set point among HIV-1 seroconverters (S11 Table), indicating that the associations we have found are unlikely to act through altered viral replication with these gene products functioning as intracellular viral restriction factors [38].

## Discussion

Our findings demonstrate that aggregates of host genetic variants, including variants with MAF<10%, can have a strong and replicated association with HIV-1 acquisition risk. Our replication of *CD101* Ig-like variants showed an HR for HIV-1 infection of 4.3 (95% CI = [2.1–8.9], p = 6.4x10^-5^); and replication of association of *UBE2V1* rs6095771 with HIV-1 acquisition risk showed an HR of 6.4 in women (95% CI = [2.1, 19.1], p = 9.5x10^-4^). Two *CD101* missense/regulatory variants reached individual statistical significance after adjustment for multiple testing, four had individual FDRs < 0.05, and a strong dose-response relationship with the frequency of unprotected sex collectively strengthened evidence that association with HIV-1 acquisition is real. While many of these variants were *individually* rare or infrequent, nearly 18% of Kenyans evaluated in the 1K Genomes Project [30] had one or more of the three most common *CD101* Ig-like HIV-1 risk variants (rs17235773, rs3754112, rs12093834). Hence, these variants or others in *CD101* and *UBE2V1* could have a substantial impact on the population-based HIV-1 infection risk.

*CD101* and *UBE2V1* are both biologically plausible candidates for influencing HIV-1 acquisition. *CD101* is expressed on CD4+ and CD8+ T-cells, dendritic cells and monocytes, [39] and appears to alter CD4+/CD25+/FOXP3+ T regulatory cell (Treg) function based on both a murine graft-versus-host disease model [40], and through IL10 secretion from human dendritic cells [41]. Monoclonal antibody ligation of *CD101* reduces T cell proliferation through a Ca^2+^ and tyrosine kinase-dependent pathway possibly by preventing translocation of nuclear factor of activated T cells (NFAT) and IL-2 production [42]. Recent experiments using a murine model of chronic colitis demonstrate that adoptive transfer of CD101^-/-^ Tregs is associated with Th17 cell proliferation and more severe colitis [43]. *CD101* expression is also strongly associated with the immune suppression function of Tregs in humans. [44] Reduced expression of CD101 on mucosal CD8+ T cells has been associated with increased tissue inflammation in studies of human intestinal [45], and pulmonary mucosa. [46] Given that local inflammation and CD4+ [47] and CD8+ [48, 49] T cell immune activation have been associated with increased HIV-1 acquisition risk, and reduced immune activation [50, 51] or immune quiescence [52, 53] have been associated with natural resistance to HIV-1 in HESN, our results support the idea that *CD101 gene* variants may modify HIV-1 heterosexual acquisition risk through altered levels of genital mucosal inflammation.

The finding that *CD101* risk variants are associated with lower plasma IL1R1 levels, but not with HIV-1 RNA set point, suggests that these variants have systemic immunological effects in the seronegative partner while not directly acting on HIV-1 replication. Recent studies in mouse models indicate that IL1 may inhibit Treg and enhance Th17 differentiation [54]. However, it is unclear at this point how variants in *CD101*, specifically those identified in *CD101* Ig-like domains, might modify either IL1 or IL1R1 levels. The *IL1R1* rs2234650 genotype has been reported to be associated with HIV-1 acquisition in infants of HIV-1 infected mothers with modification of risk by *IL1* gene family haplotypes [55]. In addition to *CD101* being associated with increased Treg function, *IL1R1* expression has been associated with increased Treg and anti-inflammatory IL10 secretion [44]. Combining these prior data with our findings, we hypothesize that *CD101* Ig-like variants reduce Treg function with associated reductions in IL1R1 levels and an enhanced pro-inflammatory environment, which leads to increased risk of HIV-1 acquisition. While efforts to test this hypothesis and to develop a more detailed understanding of *CD101* function are underway, our results suggest that targeting *CD101* activity could be a novel approach to host-directed, HIV-1 prevention.

UBE2V1 associates with TRIM5-α, a host restriction factor involved with HIV-1 capsid uncoating [56]; however, rare and uncommon *UBE2V1* variants have not previously been studied in association with HIV-1 acquisition risk. Previous GWAS without rare variant burden/aggregation tests cannot adequately assess the *UBE2V1* association observed here because of the rarity of the variants found to be associated with HIV-1 acquisition risk in this study. *UBE2V1* forms an ubiquitin-conjugating complex generating unattached polyubiquitin chains that may stimulate NF-κB activation and consequent pro-inflammatory cytokine production [56, 57]. This complements reports of reduced systemic immune activation associated with resistance to HIV-1 acquisition in Kenyan sex workers [50, 53, 58]. Of note, *UBE2V1* is one of only 23 genes differentially down-regulated by HIV-1 trans-activator of transcription (TAT), an HIV-1 protein that is required for efficient replication of the HIV-1 virus and potential escape from the host immune system [59].

Distinguishing between a group carrying variants protecting against HIV-1 acquisition versus a group carrying risk-increasing variants requires that both groups are exposed to HIV-1, i.e. to assess susceptibility to HIV-1 in any given individual, that individual must be exposed to HIV-1. This translates directly to a mathematical proof that statistical power to detect a significant association with HIV-1 acquisition risk is increased by selectively identifying individuals with sustained high levels of HIV-1 exposure. Indeed, the success of this study was dependent on our ability to quantify HIV-1 exposure with relatively high accuracy to identify exposed individuals. Given that the overall per-contact probability of heterosexual HIV-1 transmission is intrinsically low (estimated at ~1/1000 for vaginal intercourse among the study population) [60], our findings suggest that inclusion of individuals with lesser HIV-1 exposure may explain, in part, why it has been difficult to identify and/or validate genetic risk factors for HIV-1 acquisition.

The use of the extreme phenotypes design is another strength of this study. The discovery of CCR5-delta32 was based on the observation that extreme resistance phenotypes existed [61, 62] leading to case-control studies to identify the CCR5-delta32 variant among candidate genes [1–3], including extreme hemophiliac controls [1] and the others employing just two to four extreme resistant controls [2, 3]. Identifying individuals with phenotypes that represent the extremes of risk of HIV-1 acquisition has been challenging because few studies are able to assess both the behavioral and biologic dimensions (e.g. frequency of unprotected sex, and plasma HIV-1 RNA level in the HIV-1 transmitting partner) that contribute to exposure. Our use of data from both partners added accuracy to the phenotypes we used in this study—e.g., plasma HIV-1 RNA level contributed by the HIV-1 infected partner, reported frequency of unprotected sex from both partners, and epidemiologic data from the HIV-1 uninfected partner (e.g., male circumcision status) [23]. Further improvements in accurate exposure quantification could increase power even more and perhaps very extreme phenotypes can be identified that provide superb power with small samples but lead to generalizable treatments. Recent examples that underscore the value of the extreme phenotype analysis approach include discovery using a sample size of a dozen individuals of a human antibody to a malaria protein that prevents death and provides a promising new malaria vaccine target [63], and discovery of the protective effect of *PKC9* loss of function variants against cardiovascular disease in a small group of extreme individuals [64], leading to the development of PKC9 inhibitors for lowering LDL cholesterol.

Our analysis identified variants in *CD101* and *UBE2V1* associated with increased risk of HIV-1 acquisition, but we did not identify variants associated with reduced risk of HIV-1 acquisition. Statistical power is lower to identify protective variants than it is to identify risk variants (of the same magnitude but inverse) when the outcome has low incidence. Discovery of protective variants for HIV-1 infection in a population with average sexual contact and some use of protection against transmission would require extended observation time to detect differences in the survivor rates (“rates of non-seroconversion,”) while differences in rates of seroconversion of the same magnitude can be detected statistically within a shorter time period.

A limitation of these results is that confounding cannot be ruled out with certainty as the source of the associations. This is true for any observational study, though observational studies remain a fundamental part in building a step-wise scientific case for causal association for many exposures that cannot be tested experimentally in humans (including exposure to genetic variants, the epidemiological exposure being tested here), with smoking as a cause of lung cancer being a prime example. [65] We have guarded against confounding to the extent possible by evaluating for known potential confounders, including ancestry group (which likely includes HLA), BV, age, cohort, ethnic group affiliation and spatially isolated ancestry. The next general step in establishing causal association is replication by different groups and functional studies. We are currently engaged in the latter and encourage validation of our results in genetic association studies with HIV-1 exposure measurement as well as functional studies by others of the variants/genes discovered and replicated here.

In summary, we used quantitative measures of HIV-exposure to select individuals with extreme HIV-1 acquisition phenotypes and thereby optimize our power to detect genes associated with risk of HIV-1 acquisition. We identified variants, including rare variants, in *CD101* and at least one in *UBE2V1* that are significantly associated with increased HIV-1 acquisition risk. More detailed dissection of the molecular basis for this association may identify unique interventions that use these pathways to improve public health prevention of HIV-1.

## Materials and methods

### Ethics statement

We identified individuals for this study from HIV-1 serodiscordant couples recruited into three cohorts of African heterosexual HIV-1 serodiscordant couples: the Partners in Prevention HSV/HIV Transmission Study [20] (ClinicalTrials.gov number, NCT00194519), the Couples Observational Study [14], and the Partners PrEP study [22] (ClinicalTrials.gov number, NCT00557245) (S1 Table). Detailed procedures have been reported elsewhere for each of these studies [14, 20, 22]. Briefly, routine follow-up visits with both partners were scheduled at least every 3 months, with clinical, behavioral and demographic data collected. HIV-1 seroconversion (SC) was assessed by HIV-1 rapid test at the study clinic; positive rapid tests were confirmed by HIV-1 ELISA at the site laboratory, and by Western Blot in batch at the University of Washington (UW). Plasma virus sequencing performed on both partners for each couple associated with SC was used to confirm transmission linkage [66]. All participants provided written informed consent for participation in the clinical study, and samples for this genotyping study were selected from among those participants recruited at 14 sites across all three cohorts who additionally consented to host genetic studies. Relevant study documents went through ethical review and approval by the following committees:

**Ethics Committees (Local and national African study sites):**
Kenya Medical Research Institute Ethics Committee;
Kenyatta National Hospital Ethics Committee;
Kilimanjaro Christian Medical College;
Moi University Ethics Committee;
Republic of Botswana Ministry of Health;
South Africa Medicines Control Council;
Uganda National Council for Science & Technology;
Uganda National AIDS Research Committee;
Uganda Virus Research Institute;
University of Witwatersrand Ethics Committee;
University of Cape Town Institutional Review Board

**Ethics Committees (Site-affiliated international institutions):**
Harvard School of Public Health;
Indiana University Institutional Review Board;
London School of Hygiene and Tropical Medicine;
United States Centers for Disease Control and Prevention;
University of California, San Francisco Institutional Review Board;
University of Washington Institutional Review Board
The University of Washington Institutional Review Board also was the institutional review board for the UW coordinating center applications for all three studies:
University of Washington Human Subjects Division #25802
University of Washington Human Subjects Division STUDY00000172 (Formerly HSD# 32528)
University of Washington Human Subjects Division #30366

### Discovery stage participant selection

An extreme phenotypes case-control design was employed for the Discovery stage. The extreme phenotypes design provides the greatest statistical power for a fixed Discovery sample size of 100 individuals (with power increasing as the percentiles of phenotype become more extreme in the two arms). Hence, individual phenotype was a primary consideration in Discovery stage participant selection. Extreme cases here comprise individuals with relatively low estimated exposure who seroconverted during the study, especially those who converted early in the study. Extreme controls comprised individuals with high estimated risk who remained seronegative over the full observation period with follow-up for at least nine months. Highly-exposed HIV-1 exposed seronegative control individuals with longer follow-up time were considered more extreme based on cumulative exposure scores across all study visits. Cumulative exposure score to rank extremes was calculated as previously described using plasma HIV-1 RNA level of the infected partner, frequency of unprotected sex and male circumcision status [23], with the modification that all participants must have reported unprotected sex by at least one partner in the couple to be eligible for the Discovery stage sample. It is possible for an individual to have a fairly high exposure score in this model even if no unprotected sex is reported, because the original risk score is based on *empirical* estimates of risk of seroconversion given the variable values, and some participants who reported no unprotected sex did seroconvert with plasma HIV-1 genomes matching those of their infected partners. Hence, the risk among the group that *reported* “never unprotected sex” is not zero. Nevertheless, the latter risk is smaller than that for those who report unprotected sex, all else equal, and the risk rises as the proportion of reports with unprotected sex rises (S6 Fig). Seronegative individuals from couples that did not report unprotected sex were excluded for statistical power reasons: including unexposed individuals lowers the statistical power to the point where a p-value of 6.3x10^-5^ in the Replication stage (N = 261)(Table 3) becomes p = 0.03 (N = 1229) (Table 4; replication stage methods below). Given these exposure scores/conditions defining the extremeness of phenotype within the potential cases and controls, we then incorporated both gender balance within-group and pairwise ethnicity/sex matching between-groups to this design to avoid happenstance confounding by differing proportions of sex or ancestry in the relatively small samples. The potential impact of ancestry-confounding in this subpopulation was largely unknown when the Discovery stage was designed, and we opted to take this precaution against confounding.

Specifically, seroconverter (SC) “cases” were selected from the Partners in Prevention HSV/HIV Transmission Study and COS cohorts among couples with laboratory confirmed linked HIV-1 transmission [67], who were HIV-1 polymerase chain reaction (PCR) negative at enrollment and had the lowest exposure scores, conditional on relatively equal numbers of males and females. Individuals from the Partners PrEP cohort (S1 Table) were not available for Discovery stage sampling because the trial had not come to complete closure with data available for ancillary studies at that time. For selection of Discovery stage controls, we excluded from consideration: SC individuals (with either linked or unlinked transmissions), couples in which HIV-1 infected partners reported use of any antiretroviral therapy (ART), couples with no reported unprotected sex during the study and couples with less than nine months follow-up time. After these exclusions, for each selected case, all HESN of matching sex and self-reported ethnicity were identified, and from these matched individuals, the HESN individual with the highest cumulative exposure score was selected as the matching control. A total of 65 case-control pairs were identified in this manner, with identification of low exposure (extreme) cases being the highest selection priority, followed by ethnicity/sex matching for controls, followed by criteria for high exposure among the matching controls.

We quality controlled these case-control pairs for gender check, cryptic relatedness and genetic heterogeneity across multiple longitudinal whole blood DNA samples, using a custom Illumina Goldengate chip with 384 single nucleotide polymorphisms (SNPs). These test SNPs were selected as being the most predictive of ancestry clusters and individual identity from a principal component analysis (PCA) on data from a previous genome-wide association study [14] that included samples from these same cohorts. Specifically, we first performed a PCA on a pruned set of 133,991 SNPs from that GWAS that had low linkage disequilibrium. The first five PCs for this analysis were effective at distinguishing participants from East and southern Africa, by country (Kenya, Uganda, Tanzania, South Africa and Botswana) and by self-reported ethnicities reported in >2% of participants (S10 Fig). Using the first five PCs, we then assigned all participants to one of nine ancestry clusters based on model-based clustering, which has previously been shown to reduce population stratification bias. Subsequently, we used the Random Forests algorithm to identify 357 SNPs that were most predictive of geographic region (East Africa versus southern Africa) and the nine ancestry clusters. These 357 SNPs were collectively able to differentiate the ancestry clusters but were much less important for predicting ancestry than self-reported ethnicity and geographic region (S11 Fig). The final Goldengate SNP chip included the 357 ancestry SNPs along with 27 SNPs that maximized the probability that all participants had a different genotype at one or more loci in order to ensure that DNA samples came from unique individuals.

The genotyping chip was used on DNA from 65 potential case-control pairs. After eliminating samples that failed QC (1 case failed cryptic relatedness, and longitudinal samples from 2 controls suggested potential sample heterogeneity), verifying matching on ancestry cluster and identifying controls with highest cumulativeHIV-1 exposure scores over all visits, 50 case-control pairs were selected for complete genome sequencing for the Discovery analysis (24 male cases with matched controls, and 26 female cases with matched controls). When characterized by the two strongest components of the exposure score, namely mean plasma HIV-1 RNA and proportion of follow-up visits where no-condom use was reported, the Discovery stage controls were verified as sampled from the highest exposure strata (Table 1). Cases also had a median time to HIV-1 seroconversion of 11.8 months, while the median duration of follow-up without seroconversion for controls was 22.8 months.

### Discovery stage whole genome sequencing (WGS), quality control and annotation

WGS was performed by Complete Genomics, Inc (CGI), using published methodology [68], and with samples blinded as to case and control assignment. SNV calling was performed by CGI using proprietary software cgatools version 1.5.0 build 31 (dev) and human reference genome NCBI Build 37. Overall, CGI sequence quality and aggregate descriptive characteristics were similar for case and control genomes (S2 Table). Genome annotations were from CGI based on the National Center for Biotechnology Information (NCBI) refSeq database. Annotations used for this rare SNV analysis included non-synonymous protein coding sequence, 3’- and 5’-untranslated regions, and splice donor/acceptor sites with exon/intron boundaries identified through the University of California-Santa Cruz (UCSC) refFlat database. [69]

To validate WGS sequence data we identified 8 SNVs in the WGS data that lacked an rsID in dbSNP and were identified in either of the two genes targeted for replication analysis (*CD101* and *UBE2V1)*. SNVs not previously reported to dbSNP have a higher probability of being false positives than the bulk of SNVs in dbSNP, so these provide “tough” test cases for the CGI calls for validation of true positive rare variants. These SNVs were Sanger sequenced from the individual genomes originally used to generate the WGS. All 8 were successfully validated (S3 Table).

### Discovery stage statistical methods

Prior to analysis, each SNV was classified as “functional” or not, based on CGI annotation indicating that the SNV either (1) altered protein coding, (2) was at a splice-site or (3) in a UTR sequence region. Only these genic categories of functional polymorphisms were included in this analysis. Case-control comparison of SNVs aggregated by genic region (“variant burden”) was accomplished through logistic regression analyses with burden scoring derived specifically for rare variant analysis by Morris and Zeggini with the p-value based on the likelihood ratio test (their RVT1) [24] The Wald test p-value was found to be unreliable—much too conservative—for genic regions with high imbalance between cases and controls, which are exactly the situations we seek to find. Although cases and controls were matched by ethnic origin, the first 3 principal components (PC) in the logistic regression of each genic region were used to control for potential residual population stratification. To focus on less common or rare variants and higher effect sizes, the RVT1 ignores variants with MAF above a specified cutoff (>0.125 in our analysis). The MAF cut-off of 0.125, which is somewhat higher than typically considered rare, was used to allow for random variation in the observed MAF for rare SNVs in the population, as well as to allow for enrichment of rare SNVs in the sample due to the selection of extremes. Because the study participants are sub-Saharan Africans, external estimates of MAFs for the observed SNVs were not available. This testing prioritization strategy resulted in 18,354 genic regions tested (each region with an RVT1 p-value produced for the null hypothesis of no difference in variant burden within the region between cases and controls) and included a total of 284,632 functional variants in these tests. Principal components created by LD-pruning variants with MAF > 0.03 were used to adjust the RVT1 tests. Genic regions with the lowest p-values were considered for replication. These also have the lowest False Discovery Rates [70] (FDRs). Based on FDR analysis, the two most significant regions had a probability of 0.83 that at least one was a true positive (not a false discovery). These two regions were moved forward to the MIPs Replication stage based on this high probability of having a true positive result.

### Replication analysis plan and criterion for significance taking into account Discovery results

We planned and implemented a formal frequentist p-value-based criterion for declaring significance of the replication of *CD101* and *UBE2V1* genic region variants contributing to the aggregate scores in the Discovery stage. We broke the variants into smaller aggregates for the replication analysis (four aggregates for *CD101*, two for *UBE2V1*) in an *a priori* attempt to further enrich at least some of these aggregates with a higher proportion of true positive variants. This reflected the fact that Discovery stage test regions include both variants that are significantly associated with outcome (i.e., signal) and those not significantly associated with outcome (i.e., noise). If sub-groups of the originally aggregated variants can be enriched for truly associated variants having a common direction of effect, the statistical power for replication of such an enriched sub-group can be increased considerably relative to the larger, noisier aggregate [19, 28] despite the need for a multiple-test correction due to increasing the number of tested aggregated variant groups. Based on statistical reasoning, variants with large effect sizes and the same direction of effect are most likely to be driving the Discovery finding. To formulate sub-groups prior to the Replication analysis, “by-variant” association results were tabulated and examined for size and direction of effect. Confidence intervals are wide for these by-variant results due to the low MAF/low-power issue, but they are not completely uninformative. To complement these results, we also compared by-variant results to genome and protein maps of the regions (Fig 1; S4 & S5 Tables). Sixteen of 24 predicted functional variants in *CD101* had by-variant estimates with increased risk (HRs>1), which is the direction of effect for the aggregate RVT1 *CD101* result that we seek to replicate. Fourteen of these were designated as PRVs based on significance of by-variant tests (S3A Fig). These 14 *CD101* PRVs were divided into Ig-like (N = 5 PRVs), cytoplasmic (N = 5), 3’-UTR (N = 2) and splice site domain (N = 2) subgroups. Similarly, 11 functional variants in *UBE2V1* identified as PRVs (S3B Fig) divided into 5’-UTR (N = 6) and 3’-UTR (N = 5) subgroups.

Although a fairly common strategy in replication of rare variant results is to include all rare variants within a region in the replication test whether or not these variants were among those identified in the Discovery stage. For example, such a strategy would be used to study low-density lipoprotein receptor (*LDLR*) variants [27, 28] (OMIM #606945), given that the structure and function of *LDLR* is well-known, providing reasonably high confidence that novel missense or truncation RVs in particular sub-regions of the *LDLR* gene will have a deleterious association. In this study, we elected not to include in replication tests any variants seen only in the Replication stage and not in the Discovery stage for four reasons: (1) identification of individual variant contributions is important at this point to identify variation in any specific structural protein components affecting the outcome (and point toward mechanism); (2) identification of individual variant contributions for variants that are only modestly rare makes it feasible to use genotype tests for presence of individual risk variants in future studies or risk assessments; (3) addition of new variants in the tests at the Replication stage obscures the contribution of variants from the Discovery stage alone; and (4) we did not expect a large contribution from new/novel/rare variants in the Replication stage because of the sample size limitations at this point, and therefore were not concerned by potential loss of statistical power via this choice.

In addition to the primary replication tests that include subsets of variants identified in the Discovery stage (four tests planned for *CD101* and two for *UBE2V1*), we performed exploratory testing on all additional variants found in the Replication stage to take the most advantage of these findings while limiting the formal replication tests as above. All exploratory statistical test results are reported by nominal p-value and FDR to provide some quantification of evidence for association, but no formal multiple test correction is given for these findings given their often highly correlated nature (making a Bonferroni correction misleading) and due to the *post hoc* character in some cases.

### Replication stage sample selection

We measured sexual exposure behavior defining Protected-sex Index (PI) as the proportion of study visits for which only abstinence or 100% condom use was reported. Simulation studies showed that statistical power was highest when the Replication cohort included individuals with PI ≤0.6 while individuals with higher levels of protected sex were excluded. We set the Replication cohort to be the 262 baseline uninfected individuals each having PI ≤ 0.6 who remained after the Discovery stage. Due to the economy of sequencing multiple individuals using multiple inversion probe sequencing (MIPs) [33] and the intention to demonstrate proof of principle in our power calculations, we successfully sequenced an additional 986 individuals (thirteen 96-well plates). This set of 1248 individuals was balanced on sex and ethnicity relative to seroconverters and non-seroconverters at a 1:6 ratio, a typical sampling strategy. These additional 986 individuals had higher exposure scores than the average participant in the serodiscordant couple cohorts based on high plasma HIV-1 RNA levels of their infected study partner, but many reported low levels of or no unprotected sex. Therefore, this group of additional participants was not considered by itself. There were 19 individuals failing the MIPs procedure due to low DNA concentration, resulting in 1229 individuals with 261 in the Replication sample and 968 in the auxiliary sample.

### Replication stage sequencing, quality control and annotation

MIPs can be used for targeted sequence capture, followed by massively parallel sequencing of captured products. This strategy is efficient and cost-effective for sequencing multiple candidate genes in modest to large sample sets [33]. MIPs were designed to target all coding exons plus 10 additional base pairs of intron/exon flanking sequence for *CD101* (RefSeq NM_004258), and *UBE2V1* (RefSeq NM_021988). In total, 74 MIPs (Integrated DNA Technologies, Coralville, IA) were designed and pooled in equimolar ratios and a test library of 24 African control samples from Centre d’Etude du Polymorphisme Humain (CEPH) and 23 Caucasian in-house control samples was produced and evaluated. For MIPs that failed to produce the minimum required average sequence depth of 60X in the test library, their concentration in the final pool was increased according to their level of under-performance in order to increase coverage above the average threshold. The optimized MIP pool (S12 Table) was then used to generate libraries and targeted sequence from the ethnically-matched Replication Sample.

### Methods for MIPS library preparation

The MIP pool was phosphorylated with T4 Polynucleotide kinase and T4 Ligase Buffer (New England Biolabs, Ipswich, MA). The reaction was held at 37°C for 45 minutes and then de-activated at 65°C for 20 minutes. For the majority of samples (N = 1096), 100ng of genomic DNA per sample was used in a capture reaction with a 200:1 MIP to gDNA ratio. For samples with less than 100ng of DNA available (N = 228), a range of 40ng to 100ng was used in the capture. During a 24-hour reaction at 60°C, HemoKlen Taq (New England Biolabs, Ipswich, MA) was used to capture the target regions and Ampligase (Epicentre, Madison, WI) was used to circularize constructs. *E*. *coli* Exonuclease I and Exonuclease III (New England Biolabs, Ipswich, MA) were used to enzymatically clean capture reactions. The reactions were held at 37°C for 30 minutes and then de-activated at 95°C for 2 minutes. Captured products were amplified using iProof HF Master Mix (Bio-Rad, Hercules, CA) during 20–22 cycles of PCR. This PCR was performed using a generic forward primer and reverse primers containing a generic portion along with a unique 8bp molecular tag used to barcode the captured products for each sample. Between 40 and 96 barcoded samples were pooled into each library and the libraries were purified using Agencourt AMPure XP (Beckman Coulter, Indianapolis, IN) magnetic beads in the ratio 0.9:1 beads to samples. Libraries were eluted into EB buffer (Qiagen, Valencia, CA) and quantified via the Broad Range Quant-iT dsDNA Assay Kit (Life Tech, Waltham, MA) using a SpectraMax Gemini XPS Fluorometer (Molecular Devices, Sunnyvale, CA). Libraries were then pooled and sequenced on an Illumina MiSeq using 300 cycle paired end (v2) reagents (Illumina, San Diego, CA). Custom oligonucleotides complementary to sequences in the MIP constructs were used. Each run contained between 136 and 192 samples. Libraries were diluted and denatured according to Illumina’s standard procedure with final loading concentrations ranging from 8 to 10pM.

Individual fastq files were generated by the MiSeq Reporter Software (v2.5). The resulting fastq files were aligned to Hg19 with the Burrows-Wheeler Aligner (BWA v0.5.9-r16), and a multi-sample variant call file was generated using the Genome Analysis Tool Kit (GATK v2.4-9-g532efad). Annotated was performed using Variant Effect Predictor v82 (http://www.ensembl.org/info/docs/tools/vep). The average sequencing depth per sample across all sites was 198X. A total of 1229 individuals’ sequences passed quality control. Given that a subset of these samples (N = 138) had less than 100ng of total DNA and therefore could have yielded high variant missingness in these samples but with good data quality in the remainder of samples, we set the threshold of 12% missingness for excluding a variant from the Replication stage tests below (compared to a conventional GWAS threshold of 5% missingness). A total of 314 variants passed quality control including a Hardy Weinberg Equilibrium test cut-off of p>3.18x10^-5^.

### Replication stage statistical methods

Testing for association between genomic variation and HIV-1 acquisition risk in the Replication stage was performed using straightforward, standard censored data methods, since follow-up time in the three cohorts was variable with most observations being censored. This provides greater statistical power than using a subset of individuals who have reached a minimum follow-up time or the outcome to create a case-control sample, as it allows all follow-up time to contribute to the estimated risk associations. Kaplan-Meier plots and Cox proportional hazards models were used. We performed the primary replication tests by scoring the aggregate risk variable as 1, if an individual carried a minor allele for any of the variants in the aggregate, and 0 otherwise. This variable was tested for association with time-to-seroconversion via the Cox model. Risk variables were tested jointly in the Cox model, as well, and results were checked for possible confounding by sex, cohort, age, country/ethnic group (five countries with Kenya broken into three major ethnic groups plus others, a surrogate for ancestry, S4 Fig). Principal components that were available on the subset of Replication individuals from the GWAS also were checked as potential confounders/adjustment variables on this subset. Because we and others [71, 72] have found bacterial vaginosis (BV) to be associated with increased risk of sexually acquired HIV-1 infection, BV is a potential confounder if associated with either *CD101* or *UBE2V1*. BV assessment (as we have previously defined it [73]) was available on a subset of Replication individuals and was tested as a potential confounder on this subset.

### Cytokine measurements and statistical methods for variant association

Cytokine levels at baseline entry to the cohorts were measured in a nested case-control subset of individuals from a previous study [37], wherein laboratory methods are described in detail. We assessed for differences in these cytokine levels among *CD101* variant carriers and non-carriers. This analysis includes all individuals who had both baseline cytokine measurements performed and were among the individuals included in the Replication stage MIPS sequencing or Discovery stage WGS in order to determine their *CD101* variant genotypes. Three *CD101* missense variants with increased-risk in the Discovery stage data also had high increased-risk in the Replication stage data and were all in the Ig-like aggregate that replicated overall (chr1:117554421, chr1:117560058, chr1:117568500). We separated individuals with cytokine measurements into individuals with any minor allele for any of these three variants and individuals without any of the three alternate variants. Individuals in the second group might have other *CD101* variants, but it is impossible to separate individuals with certainty into those with and without any *CD101* causal variants; but this grouping will result in a *conservative* estimate of the difference between groups, as some individuals with causal *CD101* variants might be included in the group “without causal” variants and diminish the difference between groups. (That is, any misclassification here will not produce false positive results but attenuate differences instead.) We then compared cytokine measurement distributions between these two groups. Since more than half of the cytokines had median values at the limit of detection in the overall group, we compared the two groups for a difference in the 75^th^ percentile rather than the median. This test assesses for a difference in the cytokine distributions primarily using information from those values that are above the limit of detection. (Five cytokines measured in the original report [37] had 75^th^ percentile values that were at the lower limit of detection and were not included in this analysis). Specifically, logistic regression was employed with the outcome being an indicator of whether a cytokine measurement was above the overall 75^th^ percentile for both groups combined. The independent variable was the *CD101* carrier status indicator and the regression was adjusted for the panel (batch) for each measurement. Several batches had significantly different means for some cytokines, and adjusting for batch generally increased the significance of the difference between gene variant groups. Mean differences in cytokine levels also were compared between groups to ensure that the logistic regression results were consistent with differences in means, though the latter will be affected by the large number of ties at the lower limit of detection.

## Supporting information

S1 Fig Flowchart of study design. (PDF)

S2 Fig QQ-plots from Discovery analysis. A. QQ-plot for the 18,354 RVT1 genic region test p-values from the Discovery stage. The highest two points correspond to *CD101* and *UBE2V1*. B. QQ-plot for p-values from by-variant tests on chromosome 1, the location of *CD101*, for variants with MAF ≥ 0.03. The by-variant test is a special case of the RVT1 where the “aggregate” is comprised of a single variant. C. Three QQ-plots for the p-values in B, with the three groups determined by MAF. Confounding by clusters of rarer variants can manifest as differences in qq-plots by MAF category, which is not seen here.
(PNG)

S3 Fig Functional variants in *CD101* (A) and *UBE2V1* (B) observed in the Discovery stage sample and included in the RVT1 test. Points indicate the Hg37 position (horizontal axis) and by-variant–log_10_(p-value) from a Cox model for the Discovery stage sample plotted in Manhattan style. Primary Replication variants (PRVs) and Replication stage test groups are indicated by colored points for panel A) *CD101*: red—Ig-like, blue—Cytoplasmic, green—UTR-3’, and cyan—Splice site; and panel B) UBE2V1: red: UTR-5’ and blue: UTR-3’; variants designated as secondary for replication testing are shown in black.
(DOCX)

S4 Fig Adjustment of Replication stage analyses by principal components from previous GWAS or by country-ethnicity. A subset of replication individuals also were participants in an earlier GWAS and have principal components (PCs) available from the GWAS. No principal components can be constructed from the sequencing of just *CD101* and *UBE2V1* for use in adjustment of replication analyses, but the GWAS-based PCs can be used for adjustment among individuals in the Replication analysis who also were in the GWAS, and country-ethnicity can also be used as an adjustment variable to check for changes in effect size due to confounding by major ancestral group. No change in the replication results for the Replication sample of N = 261 are seen after adjustment for country-ethnicity (HR = 4.33 (p = 6.44e-05) without adjustment, and HR = 4.64 (p = 3.74x10-5) when country-ethnicity is included in the model in addition to cohort and sex.) In regard to use of PCs for adjustment, on a subset of N = 87 individuals with PCs available (and PI ≤ 0.9 in order to increase the sample size), the HR unadjusted for PCs is HR = 3.84 (p = 0.01) while HR = 4.78 (p = 0.009) after adjustment for the most significant three PCs. In other words, there is no evidence of confounding by major ancestral group.
(PNG)

S5 Fig QQ-plot for individual variant p-values for *CD101* missense variants found in the Replication stage having empirical MAF > 0.005. The four most significant variants have a False Discovery Rate < 5% and are all in Ig-like regions; three of these four are Primary Replication Variants (PRVs) found in the Discovery stage (rs17235773, rs3754112, rs12093834). The fourth, rs140567712, has an observed MAF = 0.005 (MAF = 0.006 in 1kG Kenyans) and a Combined Annotation And Depletion (CADD) score of 16.7, third highest among the *CD101* variants found in this study (S8 Table). Variant CADD scores are correlated with causal effects *(35)*. Although not perfectly predictive, variants with higher CADD scores are currently believed to be more likely to be pathogenic or result in selection.
(DOCX)

S6 Fig HR for *CD101* Ig-like variants by Protected-sex Index (PI). A. Similar to Fig 4A except only Ig-like variants with FDR < 0.05 are included in this estimation. The large number of individuals in the auxiliary sample who report no unprotected sexual intercourse with the infected partner causes a leverage point in the model and a sharp bend in the functional form of the relationship. This was taken into account by transforming the PI to log(10*PI + 1)– 1 in the dose-response model for the *CD101* Ig-like PRV score (S4 Fig), which retains the [0, 1] range but provides a better approximation to the functional form compared to including PI as a linear variable in the Cox model. B. Plot of number of couples, divided into Replication and Auxiliary cohorts, by level of protected-sex index (PI).
(DOCX)

S7 Fig Log-type transformation of Protected-sex Index (PI) used to improve the dose-response model fit for the *CD101* Ig-like variant score in the Cox model. This transformation retains the [0, 1] range of the PI for ease of coefficient/HR interpretation. The PI is the proportion of visit surveys at which only protected sex or abstinence was reported (no unprotected sex with the HIV-1 infected partner.)
(DOCX)

S8 Fig QQ-plot for significance of differences in cytokine distribution between *CD101* Ig-like risk variant carriers and non-carriers. The *CD101* carrier group includes 58 individuals with serum cytokine measurements who have at least one alternative allele at chr1:117554421 or chr1:117560058 or chr1:117568500, which are the three variants in the Ig-like Primary Replication Variants (PRV) group that had individual FDRs < 0.05 in the replication stage. The non-carrier group (N = 105) includes individuals without alternate alleles detected at any of these three *CD101* sites. P-values are for the odds of being in the fourth (highest) quartile of the cytokine distribution over both groups. The distribution of IL1R1 levels among carriers was significantly different between groups (OR = 0.19, 95% CI = [0.07, 0.54], p = 0.0017; adjusted p < 0.05)(S10 Table).
(DOCX)

S9 Fig Distributions of IL1R1 among individuals with and without Ig-like variants in the cytokine analyses. The distributions of 25 cytokines were screened for association with presence of any minor allele for the three most common of the five Ig-like primary replication variants (rs34999087, rs17235773, and rs12093834) among 163 individuals in the Augmented Replication sample plus Discovery sample who have cytokine measurements available. Association with IL1R1 distribution was significant after adjustment for multiple testing (OR = 0.19 for achieving the 75th percentile IL1R1 value, 95% CI = [0.07, 0.54], p = 1.7x10^-3^; adjusted p = 0.04), indicating significantly lower levels of IL1R1 among those with the Ig-like missense variants. Shown are the distributions of log(IL1R1) after adjustment for panel/batch for individuals in the cytokine analyses with and without these Ig-like primary replication missense variants.
(DOCX)

S10 Fig Principal component analysis (PCA) of 135,000 single nucleotide polymorphisms from a previous genome-wide association study of 798 individuals from sub-Saharan Africa, by self-reported ethnicity and model based ancestry clusters. Plots are restricted to participants from ethnicities reported by >2% of study participants. A) Parallel coordinates showing the first five scaled PCs by self-reported ethnicity. Transparent lines represent each individual. Thick lines are from smoothed lowess curves and represent the average within self-reported ethnicity groups. B) PCs 1 and 2 by self-reported ethnicity. Points from each of five countries (Kenya, Uganda, Tanzania, South Africa and Botswana) are shown in grey. C) PCs 1 and 2 by ancestry cluster determined by model based clustering of PCs 1–10.
(DOCX)

S11 Fig Analyses of 384 single nucleotide polymorphisms (SNPs) selected for custom Illumina Goldengate SNP chip. These data were used to quality control these case-control pairs for gender check, cryptic relatedness and genetic heterogeneity across multiple longitudinal whole blood DNA samples. A) Parallel coordinates showing the first five scaled principal components (PC) estimated using 384 SNPs by ancestry clusters determined using PC analysis (PC) of 133,991 SNPs. B) PCs 1 and 2 by ancestry cluster. C) Variable importance measures from Random Forest analysis of 384 SNPs to predict ancestry cluster. D) Variable importance measures from Random Forest analysis to predicted ancestry cluster using 384 SNPs, geographic region (east vs. southern Africa) and self-reported ethnicity.
(DOCX)

S1 Table Cohorts providing study samples and data. PrEP participants were available for the Replication sample but not available for selection in the Discovery stage because the trial was still ongoing at the time of Discovery sample selection.
(DOCX)

S2 Table Characteristics of Discovery stage whole genome sequences (n = 100). (DOCX)

S3 Table Sanger validation of whole genome sequencing. All variants that had no rsID in dbSNP at the time of WGA sequencing in genes moved forward for replication were Sanger sequenced from the same original DNA sample used for WGS. Given these are not previously verified sites of variation, these variants have a higher likelihood of being sequencing errors. All eight variants with the highest probability of having false-positive calls were replicated by Sanger sequencing.
(DOCX)

S4 Table *CD101* common and rare variation (Chr 1) from Discovery samples. (XLSX)

S5 Table *UBE2V1* common and rare variation (Chr 20) from Discovery samples. (XLSX)

S6 Table Characteristics of Replication and auxiliary samples. (DOCX)

S7 Table Primary Replication Variants found by MIPS in replication and augmented (Replication plus auxiliary) samples. A. *CD101*. B. *UBE2V1*.
(DOCX)

S8 Table Annotation and summary statistics for *CD101* (Chr 1) variants observed in Replication and auxiliary samples sequenced via MIPS (N = 1229). (XLSX)

S9 Table Annotation and summary statistics for *UBE2V1* (Chr 20) variants observed in Replication and auxiliary samples sequenced via MIPs (N = 1229). (XLSX)

S10 Table Cytokine distributions comparing individuals with any of the three Ig-like *CD101* variants (rs17235773, rs3754112, rs12093834) to individuals without any *CD101* PRVs. This analysis included all individuals with cytokine measurements and adjusted for cytokine panel. Odds ratios (ORs) here are defined as the ratio of odds of having a cytokine value in the top quartile given that one of these three variants is present versus the odds given that all PRVs are absent. This method of testing for distributional differences was used due to large numbers of values below the limit of detection (averages are not meaningful in this situation) along with right-skewing of higher values (making a quantile approach appropriate).
(DOCX)

S11 Table Comparison of plasma HIV-1 RNA set point of seroconverters with and without PRVs in *CD101* or *UBE2V1* (N = 158). Mean log_10_ plasma HIV-1 RNA for N = 158 individuals with versus without primary replication variants (PRVs) in *CD101* or *UBE2V1* and who HIV-1 seroconverted during study follow-up. The P-value estimate for this comparison, as well as the lower and upper bounds on the 95% confidence interval, are also shown.
(DOCX)

S12 Table MIPS primers. (DOCX)

S1 References References cited in supplementary data files. (DOCX)

S1 Data (XLSX)

## References

[1] Dean M, Carrington M, Winkler C, Huttley GA, Smith MW, Allikmets R, et al Genetic restriction of HIV-1 infection and progression to AIDS by a deletion allele of the CKR5 structural gene Science. 1996;273:1856–62. 8791590 10.1126/science.273.5283.1856

[2] Liu R, Paxton WA, Choe S, Ceradini D, Martin SR, Horuk R, et al Homozygous defect in HIV-1 coreceptor accounts for resistance of some multiply-exposed individuals to HIV-1 infection. Cell. 1996;86:367–77. 8756719 10.1016/s0092-8674(00)80110-5

[3] Samson M, Libert F, Doranz BJ, Rucker J, Liesnard C, Farber CM, et al Resistance to HIV-1 infection in caucasian individuals bearing mutant alleles of the CCR-5 chemokine receptor gene. Nature. 1996;382:722–5. doi: 10.1038/382722a0 8751444 10.1038/382722a0

[4] Fowke KR, Nagelkerke NJ, Kimani J, Simonsen JN, Anzala AO, Bwayo JJ, et al Resistance to HIV-1 infection among persistently seronegative prostitutes in Nairobi, Kenya. Lancet. 1996;348:1347–51. doi: 10.1016/S0140-6736(95)12269-2 8918278 10.1016/S0140-6736(95)12269-2

[5] Goh WC, Markee J, Akridge RE, Meldorf M, Musey L, Karchmer T, et al Protection against human immunodeficiency virus type 1 infection in persons with repeated exposure: evidence for T cell immunity in the absence of inherited CCR5 coreceptor defects. J Infect Dis. 1999;179:548–57. doi: 10.1086/314632 9952360 10.1086/314632

[6] Lo Caputo S, Trabattoni D, Vichi F, Piconi S, Lopalco L, Villa ML, et al Mucosal and systemic HIV-1-specific immunity in HIV-1-exposed but uninfected heterosexual men. AIDS. 2003;17:531–9. 12598773 10.1097/00002030-200303070-00008

[7] Horton RE, McLaren PJ, Fowke K, Kimani J, Ball TB. Cohorts for the study of HIV-1-exposed but uninfected individuals: benefits and limitations. J Infect Dis. 2010;202 Suppl 3:S377–81. doi: 10.1086/655971 20887228 10.1086/655971

[8] Johns Hopkins University. https://omim.org/.

[9] Loeuillet C, Deutsch S, Ciuffi A, Robyr D, Taffe P, Munoz M, et al In vitro whole-genome analysis identifies a susceptibility locus for HIV-1. PLoS Biol. 2008;6:e32 doi: 10.1371/journal.pbio.0060032 18288889 10.1371/journal.pbio.0060032 PMC2245987

[10] Johnson EO, Hancock DB, Gaddis NC, Levy JL, Page G, Novak SP, et al Novel genetic locus implicated for HIV-1 acquisition with putative regulatory links to HIV replication and infectivity: a genome-wide association study. PLoS One. 2015;10:e0118149 doi: 10.1371/journal.pone.0118149 25786224 10.1371/journal.pone.0118149 PMC4364715

[11] Joubert BR, Lange EM, Franceschini N, Mwapasa V, North KE, Meshnick SR, et al A whole genome association study of mother-to-child transmission of HIV in Malawi. Genome Med. 2010;2:17 doi: 10.1186/gm138 20487506 10.1186/gm138 PMC2873795

[12] Lane J, McLaren PJ, Dorrell L, Shianna KV, Stemke A, Pelak K, et al A genome-wide association study of resistance to HIV infection in highly exposed uninfected individuals with hemophilia A. Hum Mol Genet. 2013;22:1903–10. doi: 10.1093/hmg/ddt033 23372042 10.1093/hmg/ddt033 PMC3613165

[13] Limou S, Delaneau O, van Manen D, An P, Sezgin E, Le Clerc S, et al Multicohort genomewide association study reveals a new signal of protection against HIV-1 acquisition. J Infect Dis. 2012;205:1155–62. doi: 10.1093/infdis/jis028 22362864 10.1093/infdis/jis028 PMC3295605

[14] Lingappa JR, Petrovski S, Kahle E, Fellay J, Shianna K, McElrath MJ, et al Genomewide association study for determinants of HIV-1 acquisition and viral set point in HIV-1 serodiscordant couples with quantified virus exposure. PloS One. 2011;6:e28632 doi: 10.1371/journal.pone.0028632 22174851 10.1371/journal.pone.0028632 PMC3236203

[15] Luo M, Sainsbury J, Tuff J, Lacap PA, Yuan XY, Hirbod T, et al A genetic polymorphism of FREM1 is associated with resistance against HIV infection in the Pumwani sex worker cohort. J Virology. 2012;86:11899–905. doi: 10.1128/JVI.01499-12 22915813 10.1128/JVI.01499-12 PMC3486297

[16] McLaren PJ, Coulonges C, Ripke S, van den Berg L, Buchbinder S, Carrington M, et al Association Study of Common Genetic Variants and HIV-1 Acquisition in 6,300 Infected Cases and 7,200 Controls. PLoS Pathogens. 2013;9:e1003515 doi: 10.1371/journal.ppat.1003515 23935489 10.1371/journal.ppat.1003515 PMC3723635

[17] Petrovski S, Fellay J, Shianna KV, Carpenetti N, Kumwenda J, Kamanga G, et al Common human genetic variants and HIV-1 susceptibility: a genome-wide survey in a homogeneous African population. AIDS. 2011;25:513–8. doi: 10.1097/QAD.0b013e328343817b 21160409 10.1097/QAD.0b013e328343817b PMC3150594

[18] McCarthy MI, Abecasis GR, Cardon LR, Goldstein DB, Little J, Ioannidis JP, et al Genome-wide association studies for complex traits: consensus, uncertainty and challenges. Nat Rev Genet. 2008;9:356–69. doi: 10.1038/nrg2344 18398418 10.1038/nrg2344

[19] Besseling J, Hovingh GK, Huijgen R, Kastelein JJ, Hutten BA. Statins in Familial Hypercholesterolemia: Consequences for Coronary Artery Disease and All-Cause Mortality. J Am Coll Cardiol. 2016;68:252–60. doi: 10.1016/j.jacc.2016.04.054 27417002 10.1016/j.jacc.2016.04.054

[20] Celum C, Wald A, Lingappa JR, Magaret AS, Wang RS, Mugo N, et al Acyclovir and Transmission of HIV-1 from Persons Infected with HIV-1 and HSV-2. N Engl J Med. 2010;362:427–39. doi: 10.1056/NEJMoa0904849 20089951 10.1056/NEJMoa0904849 PMC2838503

[21] Lingappa JR, Kahle E, Mugo N, Mujugira A, Magaret A, Baeten J, et al Characteristics of HIV-1 discordant couples enrolled in a trial of HSV-2 suppression to reduce HIV-1 transmission: the partners study. PLoS ONE. 2009;4:e5272 doi: 10.1371/journal.pone.0005272 19404392 10.1371/journal.pone.0005272 PMC2671170

[22] Baeten JM, Donnell D, Ndase P, Mugo NR, Campbell JD, Wangisi J, et al Antiretroviral prophylaxis for HIV prevention in heterosexual men and women. N Engl J Med. 2012;367:399–410. doi: 10.1056/NEJMoa1108524 22784037 10.1056/NEJMoa1108524 PMC3770474

[23] Mackelprang RD, Baeten JM, Donnell D, Celum C, Farquhar C, de Bruyn G, et al Quantifying Ongoing HIV-1 Exposure in HIV-1-Serodiscordant Couples to Identify Individuals With Potential Host Resistance to HIV-1. J of Infect Ds. 2012;206:1299–308. doi: 10.1093/infdis/jis480 22926009 10.1093/infdis/jis480 PMC3448964

[24] Morris AP, Zeggini E. An evaluation of statistical approaches to rare variant analysis in genetic association studies. Genetic epidemiology. 2010;34:188–93. doi: 10.1002/gepi.20450 19810025 10.1002/gepi.20450 PMC2962811

[25] Storey JD, Tibshirani R. Statistical significance for genomewide studies. Proc Natl Acad Sci U S A. 2003;100:9440–5. doi: 10.1073/pnas.1530509100 12883005 10.1073/pnas.1530509100 PMC170937

[26] Mathieson I, McVean G. Differential confounding of rare and common variants in spatially structured populations. Nat Genet. 2012;44:243–6. doi: 10.1038/ng.1074 22306651 10.1038/ng.1074 PMC3303124

[27] Do R, Stitziel NO, Won HH, Jorgensen AB, Duga S, Angelica Merlini P, et al Exome sequencing identifies rare LDLR and APOA5 alleles conferring risk for myocardial infarction. Nature. 2015;518:102–6. doi: 10.1038/nature13917 25487149 10.1038/nature13917 PMC4319990

[28] Thormaehlen AS, Schuberth C, Won HH, Blattmann P, Joggerst-Thomalla B, Theiss S, et al Systematic cell-based phenotyping of missense alleles empowers rare variant association studies: a case for LDLR and myocardial infarction. PLoS Genet. 2015;11:e1004855 doi: 10.1371/journal.pgen.1004855 25647241 10.1371/journal.pgen.1004855 PMC4409815

[29] Ruegg CL, Rivas A, Madani ND, Zeitung J, Laus R, Engleman EG. V7, a novel leukocyte surface protein that participates in T cell activation. II. Molecular cloning and characterization of the V7 gene. J Immunol. 1995;154:4434–43. 7722300

[30] Genomes Project Consortium, Abecasis GR, Auton A, Brooks LD, DePristo MA, Durbin RM, et al An integrated map of genetic variation from 1,092 human genomes. Nature. 2012;491:56–65. doi: 10.1038/nature11632 23128226 10.1038/nature11632 PMC3498066

[31] Exome Sequencing Project. Exome Variant Server, NHLBI GO Exome Sequencing Project (ESP) [cited 2016 September]. http://evs.gs.washington.edu/EVS/).

[32] Lek M, Karczewski KJ, Minikel EV, Samocha KE, Banks E, Fennell T, et al Analysis of protein-coding genetic variation in 60,706 humans. Nature. 2016;536:285–91. doi: 10.1038/nature19057 27535533 10.1038/nature19057 PMC5018207

[33] O'Roak BJ, Vives L, Fu W, Egertson JD, Stanaway IB, Phelps IG, et al Multiplex targeted sequencing identifies recurrently mutated genes in autism spectrum disorders. Science. 2012;338:1619–22. doi: 10.1126/science.1227764 23160955 10.1126/science.1227764 PMC3528801

[34] Yates A, Akanni W, Amode MR, Barrell D, Billis K, Carvalho-Silva D, et al Ensembl 2016. Nucleic Acids Res. 2016;44:D710–6. doi: 10.1093/nar/gkv1157 26687719 10.1093/nar/gkv1157 PMC4702834

[35] Kircher M, Witten DM, Jain P, O'Roak BJ, Cooper GM, Shendure J. A general framework for estimating the relative pathogenicity of human genetic variants. Nat Genet. 2014;46:310–5. doi: 10.1038/ng.2892 24487276 10.1038/ng.2892 PMC3992975

[36] Naranbhai V, Abdool Karim SS, Altfeld M, Samsunder N, Durgiah R, Sibeko S, et al Innate immune activation enhances hiv acquisition in women, diminishing the effectiveness of tenofovir microbicide gel. J Infect Dis. 2012;206:993–1001. doi: 10.1093/infdis/jis465 22829639 10.1093/infdis/jis465 PMC3501691

[37] Kahle EM, Bolton M, Hughes JP, Donnell D, Celum C, Lingappa JR, et al Plasma cytokine levels and risk of HIV type 1 (HIV-1) transmission and acquisition: a nested case-control study among HIV-1-serodiscordant couples. J Infect Dis. 2015;211:1451–60. doi: 10.1093/infdis/jiu621 25389306 10.1093/infdis/jiu621 PMC4447828

[38] Colomer-Lluch M, Gollahon LS, Serra-Moreno R. Anti-HIV Factors: Targeting Each Step of HIV's Replication Cycle. Curr HIV Res. 2016;14:175–82. 26957194 10.2174/1570162x14999160224094621

[39] Rivas A, Ruegg CL, Zeitung J, Laus R, Warnke R, Benike C, et al V7, a novel leukocyte surface protein that participates in T cell activation. I. Tissue distribution and functional studies. J Immunol. 1995;154:4423–33. 7722299

[40] Fernandez I, Zeiser R, Karsunky H, Kambham N, Beilhack A, Soderstrom K, et al CD101 surface expression discriminates potency among murine FoxP3+ regulatory T cells. J Immunol. 2007;179:2808–14. 17709494 10.4049/jimmunol.179.5.2808

[41] Bouloc A, Bagot M, Delaire S, Bensussan A, Boumsell L. Triggering CD101 molecule on human cutaneous dendritic cells inhibits T cell proliferation via IL-10 production. Eur J Immunol. 2000;30:3132–9. 11093127 10.1002/1521-4141(200011)30:11<3132::AID-IMMU3132>3.0.CO;2-E

[42] Soares LR, Tsavaler L, Rivas A, Engleman EG. V7 (CD101) ligation inhibits TCR/CD3-induced IL-2 production by blocking Ca2+ flux and nuclear factor of activated T cell nuclear translocation. J Immunol. 1998;161:209–17. 9647226

[43] Schey R, Dornhoff H, Baier JL, Purtak M, Opoka R, Koller AK, et al CD101 inhibits the expansion of colitogenic T cells. Mucosal Immunol. 2016 doi: 10.1038/mi.2015.139 26813346 10.1038/mi.2015.139 PMC4963314

[44] Hua J, Davis SP, Hill JA, Yamagata T. Diverse Gene Expression in Human Regulatory T Cell Subsets Uncovers Connection between Regulatory T Cell Genes and Suppressive Function. J Immunol. 2015;195:3642–53. doi: 10.4049/jimmunol.1500349 26371251 10.4049/jimmunol.1500349

[45] Brimnes J, Allez M, Dotan I, Shao L, Nakazawa A, Mayer L. Defects in CD8+ regulatory T cells in the lamina propria of patients with inflammatory bowel disease. J Immunol. 2005;174:5814–22. 15843585 10.4049/jimmunol.174.9.5814

[46] Kumar BV, Ma W, Miron M, Granot T, Guyer RS, Carpenter DJ, et al Human Tissue-Resident Memory T Cells Are Defined by Core Transcriptional and Functional Signatures in Lymphoid and Mucosal Sites. Cell Rep. 2017;20:2921–34. doi: 10.1016/j.celrep.2017.08.078 28930685 10.1016/j.celrep.2017.08.078 PMC5646692

[47] Masson L, Mlisana K, Little F, Werner L, Mkhize NN, Ronacher K, et al Defining genital tract cytokine signatures of sexually transmitted infections and bacterial vaginosis in women at high risk of HIV infection: a cross-sectional study. Sex Transm Infect. 2014;90:580–7. doi: 10.1136/sextrans-2014-051601 25107710 10.1136/sextrans-2014-051601

[48] Kalia V, Penny LA, Yuzefpolskiy Y, Baumann FM, Sarkar S. Quiescence of Memory CD8(+) T Cells Is Mediated by Regulatory T Cells through Inhibitory Receptor CTLA-4. Immunity. 2015;42:1116–29. 26084026 10.1016/j.immuni.2015.05.023

[49] Kuebler PJ, Mehrotra ML, Shaw BI, Leadabrand KS, Milush JM, York VA, et al Persistent HIV Type 1 Seronegative Status Is Associated With Lower CD8+ T-Cell Activation. J Infect Dis. 2015 doi: 10.1093/infdis/jiv425 26310308 10.1093/infdis/jiv425 PMC4721903

[50] McLaren PJ, Ball TB, Wachihi C, Jaoko W, Kelvin DJ, Danesh A, et al HIV-exposed seronegative commercial sex workers show a quiescent phenotype in the CD4+ T cell compartment and reduced expression of HIV-dependent host factors. J Infect Dis. 2010;202 Suppl 3:S339–44. doi: 10.1086/655968 20887221 10.1086/655968

[51] Lajoie J, Juno J, Burgener A, Rahman S, Mogk K, Wachihi C, et al A distinct cytokine and chemokine profile at the genital mucosa is associated with HIV-1 protection among HIV-exposed seronegative commercial sex workers. Mucosal Immunol. 2012 doi: 10.1038/mi.2012.7 22318497 10.1038/mi.2012.7

[52] Card CM, Ball TB, Fowke KR. Immune quiescence: a model of protection against HIV infection. Retrovirology. 2013;10:141 doi: 10.1186/1742-4690-10-141 24257114 10.1186/1742-4690-10-141 PMC3874678

[53] Card CM, McLaren PJ, Wachihi C, Kimani J, Plummer FA, Fowke KR. Decreased Immune Activation in Resistance to HIV-1 Infection Is Associated with an Elevated Frequency of CD4(+)CD25(+)FOXP3(+) Regulatory T Cells. J Infect Dis. 2009;199:1318–22. 19301980 10.1086/597801

[54] Ikeda S, Saijo S, Murayama MA, Shimizu K, Akitsu A, Iwakura Y. Excess IL-1 signaling enhances the development of Th17 cells by downregulating TGF-beta-induced Foxp3 expression. J Immunol. 2014;192:1449–58. doi: 10.4049/jimmunol.1300387 24431229 10.4049/jimmunol.1300387

[55] Ahir S, Chaudhari D, Chavan V, Samant-Mavani P, Nanavati R, Mehta P, et al Polymorphisms in IL-1 gene cluster and its association with the risk of perinatal HIV transmission, in an Indian cohort. Immunol Lett. 2013;153:1–8. doi: 10.1016/j.imlet.2013.05.008 23769826 10.1016/j.imlet.2013.05.008

[56] Pertel T, Hausmann S, Morger D, Zuger S, Guerra J, Lascano J, et al TRIM5 is an innate immune sensor for the retrovirus capsid lattice. Nature. 2011;472:361–5. doi: 10.1038/nature09976 21512573 10.1038/nature09976 PMC3081621

[57] Xia ZP, Sun L, Chen X, Pineda G, Jiang X, Adhikari A, et al Direct activation of protein kinases by unanchored polyubiquitin chains. Nature. 2009;461:114–9. doi: 10.1038/nature08247 19675569 10.1038/nature08247 PMC2747300

[58] Ball TB, Ji H, Kimani J, McLaren P, Marlin C, Hill AV, et al Polymorphisms in IRF-1 associated with resistance to HIV-1 infection in highly exposed uninfected Kenyan sex workers. AIDS. 2007;21:1091–101. doi: 10.1097/QAD.0b013e3280ef6ae1 17502719 10.1097/QAD.0b013e3280ef6ae1

[59] Lin CW, Kuo JH, Jan MS. The global gene-expression profiles of U-937 human macrophages treated with Tat peptide and Tat-FITC conjugate. J Drug Target. 2012;20:515–23. doi: 10.3109/1061186X.2012.693496 22632162 10.3109/1061186X.2012.693496

[60] Hughes JP, Baeten JM, Lingappa JR, Magaret AS, Wald A, de Bruyn G, et al Determinants of per-coital-act HIV-1 infectivity among African HIV-1-serodiscordant couples. J Infect Dis. 2012;205:358–65. doi: 10.1093/infdis/jir747 22241800 10.1093/infdis/jir747 PMC3256946

[61] Dragic T, Litwin V, Allaway GP, Martin SR, Huang Y, Nagashima KA, et al HIV-1 entry into CD4+ cells is mediated by the chemokine receptor CC-CKR-5. Nature. 1996;381:667–73. doi: 10.1038/381667a0 8649512 10.1038/381667a0

[62] Paxton WA, Martin SR, Tse D, O'Brien TR, Skurnick J, VanDevanter NL, et al Relative resistance to HIV-1 infection of CD4 lymphocytes from persons who remain uninfected despite multiple high-risk sexual exposure. Nat Med. 1996;2:412–7. 8597950 10.1038/nm0496-412

[63] Raj DK, Nixon CP, Nixon CE, Dvorin JD, DiPetrillo CG, Pond-Tor S, et al Antibodies to PfSEA-1 block parasite egress from RBCs and protect against malaria infection. Science. 2014;344:871–7. doi: 10.1126/science.1254417 24855263 10.1126/science.1254417 PMC4184151

[64] Cohen J, Pertsemlidis A, Kotowski IK, Graham R, Garcia CK, Hobbs HH. Low LDL cholesterol in individuals of African descent resulting from frequent nonsense mutations in PCSK9. Nat Genet. 2005;37:161–5. doi: 10.1038/ng1509 15654334 10.1038/ng1509

[65] Rothman KJ, Greenland S. Causation and causal inference in epidemiology. Am J Public Health. 2005;95 Suppl 1:S144–50. doi: 10.2105/AJPH.2004.059204 16030331 10.2105/AJPH.2004.059204

[66] Campbell M, Mullins J, Hughes J, Celum C, Wong K, Raugi D, et al Identifying the transmitted virus: viral linkage in HIV-1 seroconverters and their partners in an HIV-1 prevention clinical trial. PLoS One. 2011;6:e16986. 21399681 10.1371/journal.pone.0016986 PMC3047537

[67] Campbell MS, Mullins JI, Hughes JP, Celum C, Wong KG, Raugi DN, et al Viral linkage in HIV-1 seroconverters and their partners in an HIV-1 prevention clinical trial. PLoS One. 2011;6:e16986 doi: 10.1371/journal.pone.0016986 21399681 10.1371/journal.pone.0016986 PMC3047537

[68] Drmanac R, Sparks AB, Callow MJ, Halpern AL, Burns NL, Kermani BG, et al Human genome sequencing using unchained base reads on self-assembling DNA nanoarrays. Science. 2010;327:78–81. doi: 10.1126/science.1181498 19892942 10.1126/science.1181498

[69] Genome Bioinformatics Group. GenePred Table format 2015 [cited 2015 11 July 2015]. Table format commonly used for gene prediction tracks in the Genome Browser]. http://genome.ucsc.edu/FAQ/FAQformat.html#format9.

[70] Storey JD. A Direct Approach to False Discovery Rates. J Royal Statistical Society Series B (Methodological). 2002;64:479–98. doi: 10.1111/1467-9868.00346

[71] Atashili J, Poole C, Ndumbe PM, Adimora AA, Smith JS. Bacterial vaginosis and HIV acquisition: a meta-analysis of published studies. AIDS. 2008;22:1493–501. doi: 10.1097/QAD.0b013e3283021a37 18614873 10.1097/QAD.0b013e3283021a37 PMC2788489

[72] Ngayo MO, Bukusi EA, Spiegel C, Mwangi J, Maina M, Lingappa J, et al. Association of abnormal vaginal flora with male-to-female HIV-1 transmission among HIV-1 discordant couples in sub-Saharan Africa. 6th IAS Conference on HIV Pathogenesis, Treatment and Prevention; July 17–20, 2011; Rome, Italy2011.

[73] Cohen CR, Lingappa JR, Baeten JM, Ngayo MO, Spiegel CA, Hong T, et al Bacterial Vaginosis Associated with Increased Risk of Female-to-Male HIV-1 Transmission: A Prospective Cohort Analysis among African Couples. PLoS Med. 2012;9:e1001251 doi: 10.1371/journal.pmed.1001251 22745608 10.1371/journal.pmed.1001251 PMC3383741

