# Correction: Whole genome sequencing of extreme phenotypes identifies variants in *CD101* and *UBE2V1* associated with increased risk of sexually acquired HIV-1

**DOI:** 10.1371/journal.ppat.1007588

**Published:** 2019-02-11

**Authors:** Romel D. Mackelprang, Michael J. Bamshad, Jessica X. Chong, Xuanlin Hou, Kati J. Buckingham, Kathryn Shively, Guy deBruyn, Nelly R. Mugo, James I. Mullins, M. Juliana McElrath, Jared M. Baeten, Connie Celum, Mary J. Emond, Jairam R. Lingappa, for the Partners in Prevention HSV/HIV Transmission Study and the Partners PrEP Study Teams

The cytokine IL1R1 appears incorrectly throughout the article. The correct cytokine name should be IL1RN, and the authors have updated the third paragraph of the Discussion section. The corrected paragraph is as follows:

We found that *CD101* risk variants are associated with lower plasma IL-1RN (interleukin-1 receptor antagonist protein) levels in HIV-1 uninfected persons, but not with altered HIV-1 RNA set point in HIV-1 seroconverters. This suggests that these variants have systemic immunological effects in the HIV-1 seronegative partner while not directly altering HIV-1 replication. IL-1RN antagonizes the pro-inflammatory effect of IL-1 by blocking IL-1 binding to its receptors. (Palomo, et al.) Recent studies in a mouse *IL-1rn*^*-/-*^ model indicate that excess IL-1 signaling may inhibit Treg and enhance Th17 differentiation [54]. However, it is unclear at this point where in the IL-1 pathway variants in *CD101*, specifically those identified in *CD101* Ig-like domains, might impact either IL-1 or IL-1RN levels. If *CD101* Ig-like variants primarily act at the level of reducing release of IL-1RN, this could leave circulating IL-1 unantagonized and therefore increase levels of Th17-associated peripheral inflammation. Alternatively, *CD101* Ig-like variants may act directly to reduce IL-1 release; which may, in turn, result in a homeostatic reduction in IL-1RN levels. While efforts proceed to better understand the mechanism by which Ig-like variants alter *CD101* function, our results suggest that development of methods to target *CD101* activity could provide a novel approach to host-directed, HIV-1 prevention.

Additionally, the authors have added a reference in the second sentence of the corrected paragraph. The reference is: Palomo J, Dietrich D, Martin P, Palmer G, Gabay C. The interleukin (IL)-1 cytokine family—Balance between agonists and antagonists in inflammatory diseases. Cytokine 2015; 76(1):25–37

In S8 Fig, the cytokine IL1R1 appears incorrectly. The correct cytokine name should be IL1RN. Please see the corrected S8 Fig here.

Additionally, there are several instances of the above error in the captions for S8 Fig and S9 Fig. Please see the complete, correct S8 Fig and S9 Fig captions here.

## Supporting information

S8 Fig QQ-plot for significance of differences in cytokine distribution between *CD101* Ig-like risk variant carriers and non-carriers. The *CD101* carrier group includes 58 individuals with serum cytokine measurements who have at least one alternative allele at chr1:117554421 or chr1:117560058 or chr1:117568500, which are the three variants in the Ig-like Primary Replication Variants (PRV) group that had individual FDRs < 0.05 in the replication stage. The non-carrier group (N = 105) includes individuals without alternate alleles detected at any of these three *CD101* sites. P-values are for the odds of being in the fourth (highest) quartile of the cytokine distribution over both groups. The distribution of IL1RN levels among carriers was significantly different between groups (OR = 0.19, 95% CI = [0.07, 0.54], p = 0.0017; adjusted p < 0.05)
(PNG)

S9 Fig Distributions of IL1RN among individuals with and without Ig-like variants in the cytokine analyses. The distributions of 25 cytokines were screened for association with presence of any minor allele for the three most common of the five Ig-like primary replication variants (rs34999087, rs17235773, and rs12093834) among 163 individuals in the Augmented Replication sample plus Discovery sample who have cytokine measurements available. Association with IL1RN distribution was significant after adjustment for multiple testing (OR = 0.19 for achieving the 75th percentile IL1RN value, 95% CI = [0.07, 0.54], p = 1.7x10^-3^; adjusted p = 0.04), indicating significantly lower levels of IL1RN among those with the Ig-like missense variants. Shown are the distributions of log(IL1RN) after adjustment for panel/batch for individuals in the cytokine analyses with and without these Ig-like primary replication missense variants.
(PNG)
