# Supplementary figures and images for: Whole genome sequencing of extreme phenotypes identifies variants in CD101 and UBE2V1 associated with increased risk of sexually acquired HIV-1
Source: PLoS Pathog. 2017 Nov 6;13(11):e1006703. doi: 10.1371/journal.ppat.1006703 (PMC5690691; doi:10.1371/journal.ppat.1006703)

## S1 Figure: Flowchart of study design

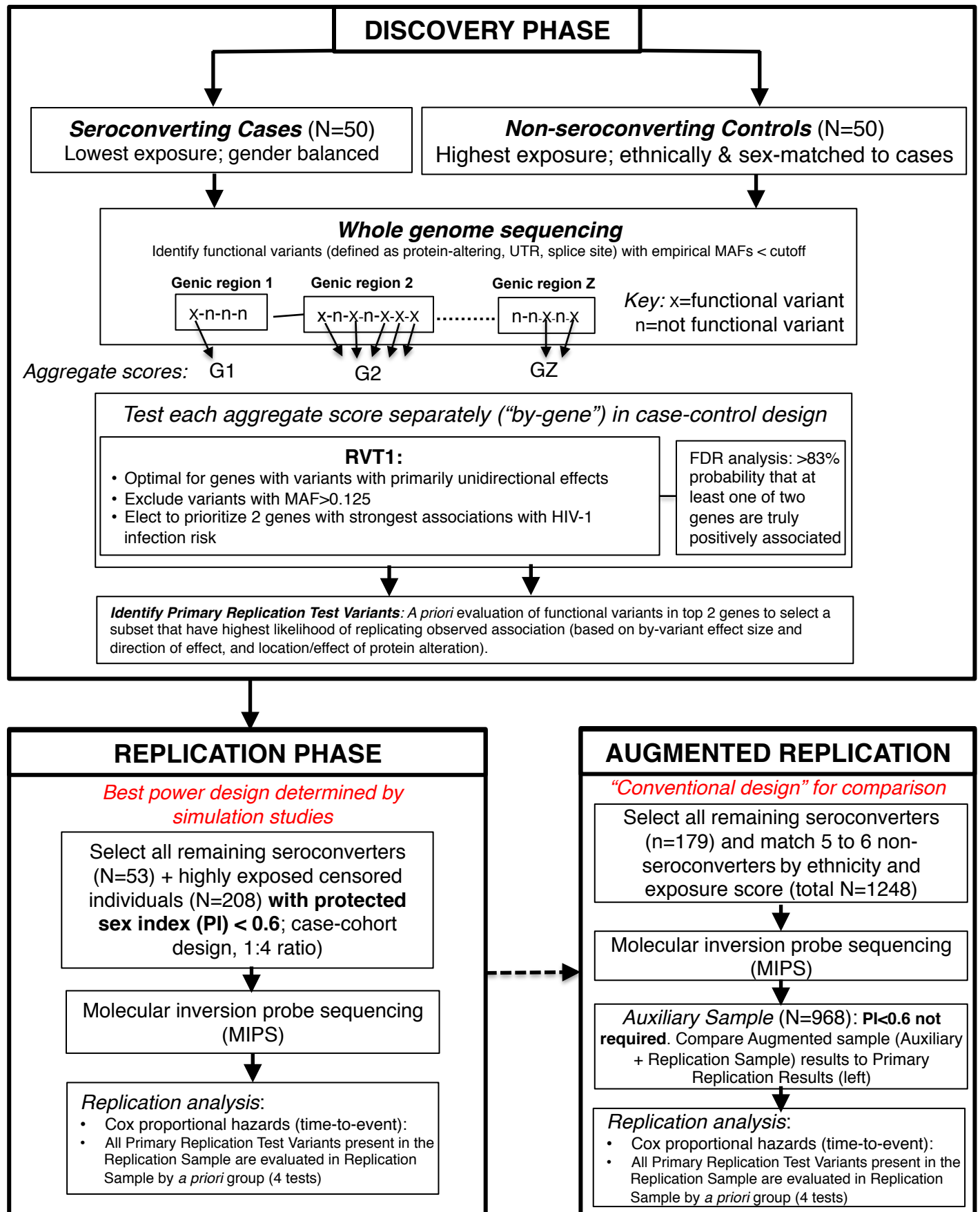

Supplement: S1 Fig — (PDF) [file ppat.1006703.s001.pdf]

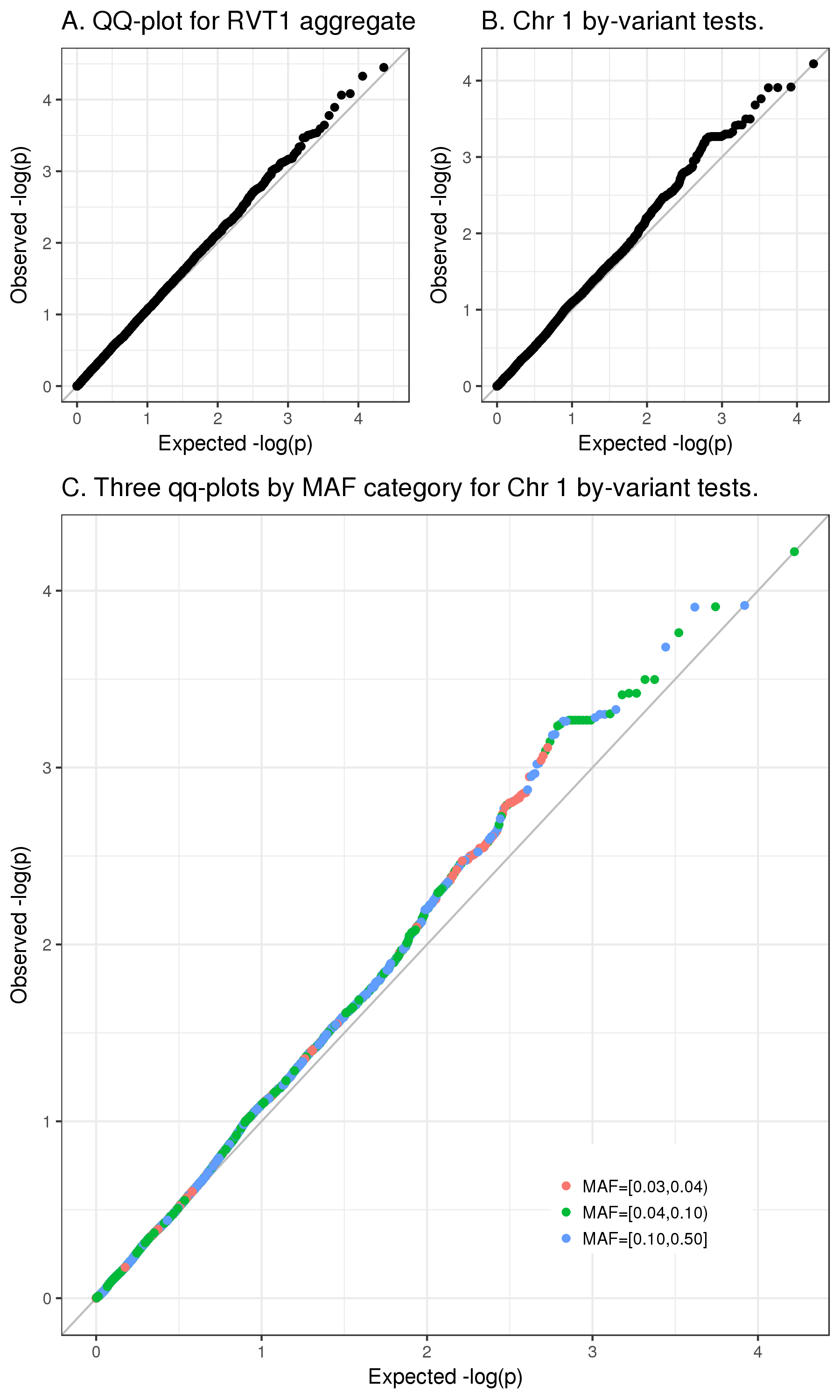

Supplement: S2 Fig — A. QQ-plot for the 18,354 RVT1 genic region test p-values from the Discovery stage. The highest two points correspond to CD101 and UBE2V1. B. QQ-plot for p-values from by-variant tests on chromosome 1, the location of CD101, for variants with MAF ≥ 0.03. The by-variant test is a special case of the RVT1 where the “aggregate” is comprised of a single variant. C. Three QQ-plots for the p-values in B, with the three groups determined by MAF. Confounding by clusters of rarer variants can manifest as differences in qq-plots by MAF category, which is not seen here. (PNG) [file ppat.1006703.s002.png]

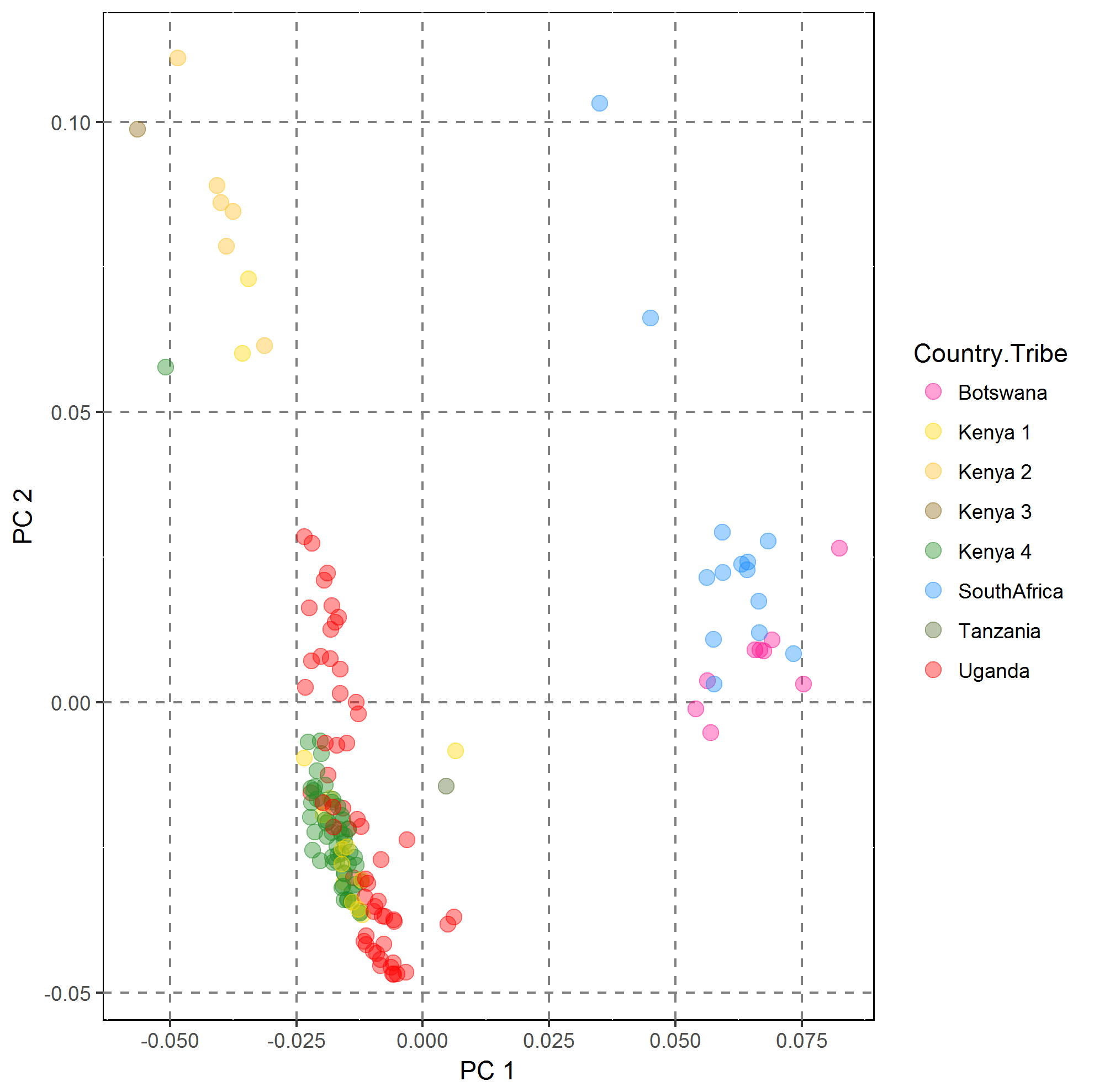

Supplement: S4 Fig — A subset of replication individuals also were participants in an earlier GWAS and have principal components (PCs) available from the GWAS. No principal components can be constructed from the sequencing of just CD101 and UBE2V1 for use in adjustment of replication analyses, but the GWAS-based PCs can be used for adjustment among individuals in the Replication analysis who also were in the GWAS, and country-ethnicity can also be used as an adjustment variable to check for changes in effect size due to confounding by major ancestral group. No change in the replication results for the Replication sample of N = 261 are seen after adjustment for country-ethnicity (HR = 4.33 (p = 6.44e-05) without adjustment, and HR = 4.64 (p = 3.74x10^-5^) when country-ethnicity is included in the model in addition to cohort and sex.) In regard to use of PCs for adjustment, on a subset of N = 87 individuals with PCs available (and PI ≤ 0.9 in order to increase the sample size), the HR unadjusted for PCs is HR = 3.84 (p = 0.01) while HR = 4.78 (p = 0.009) after adjustment for the most significant three PCs. In other words, there is no evidence of confounding by major ancestral group. (PNG) [file ppat.1006703.s004.png]

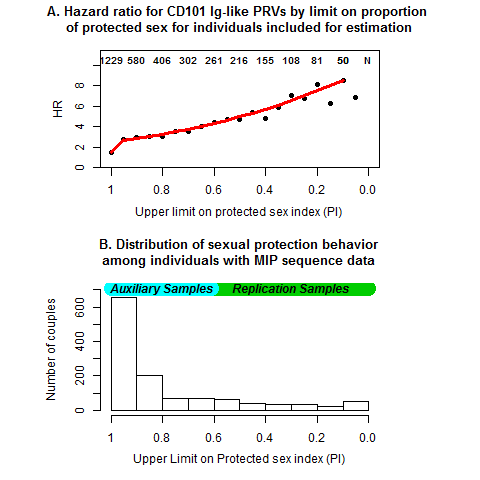


**S6 Fig: HR for *CD101* Ig-like variants by PI**

Supplement: S6 Fig — A. Similar to Fig 4A except only Ig-like variants with FDR < 0.05 are included in this estimation. The large number of individuals in the auxiliary sample who report no unprotected sexual intercourse with the infected partner causes a leverage point in the model and a sharp bend in the functional form of the relationship. This was taken into account by transforming the PI to log(10*PI + 1)– 1 in the dose-response model for the CD101 Ig-like PRV score (S4 Fig), which retains the [0, 1] range but provides a better approximation to the functional form compared to including PI as a linear variable in the Cox model. B. Plot of number of couples, divided into Replication and Auxiliary cohorts, by level of protected-sex index (PI). (DOCX) [file ppat.1006703.s006.docx]
