# Supplementary material for: Whole genome sequencing of extreme phenotypes identifies variants in CD101 and UBE2V1 associated with increased risk of sexually acquired HIV-1
Source: PLoS Pathog. 2017 Nov 6;13(11):e1006703. doi: 10.1371/journal.ppat.1006703 (PMC5690691; doi:10.1371/journal.ppat.1006703)
Supplement: S3 Fig — Points indicate the Hg37 position (horizontal axis) and by-variant–log10(p-value) from a Cox model for the Discovery stage sample plotted in Manhattan style. Primary Replication variants (PRVs) and Replication stage test groups are indicated by colored points for panel A) CD101: red—Ig-like, blue—Cytoplasmic, green—UTR-3’, and cyan—Splice site; and panel B) UBE2V1: red: UTR-5’ and blue: UTR-3’; variants designated as secondary for replication testing are shown in black. (DOCX) [file ppat.1006703.s003.docx]

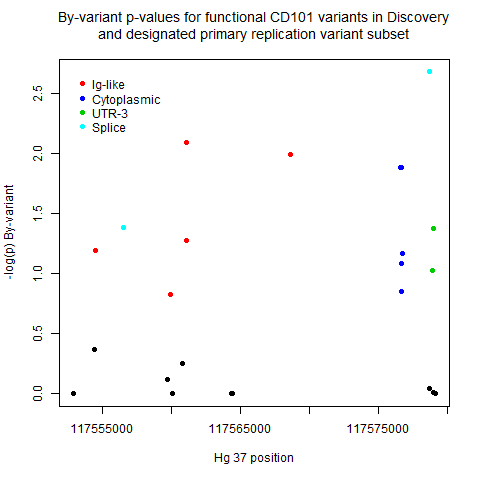

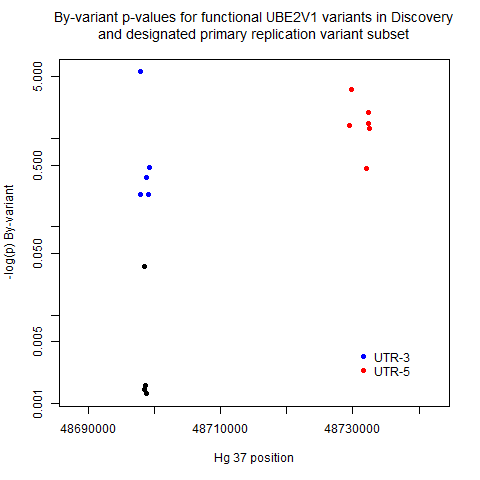


A. *CD101* B. *UBE2V1*

B.

**S3 Fig: Functional variants in *CD101* (A) and *UBE2V1* (B) observed in the Discovery stage sample and included in the RVT1 test.**
