# Supplementary material for: Whole genome sequencing of extreme phenotypes identifies variants in CD101 and UBE2V1 associated with increased risk of sexually acquired HIV-1
Source: PLoS Pathog. 2017 Nov 6;13(11):e1006703. doi: 10.1371/journal.ppat.1006703 (PMC5690691; doi:10.1371/journal.ppat.1006703)
Supplement: S5 Fig — The four most significant variants have a False Discovery Rate < 5% and are all in Ig-like regions; three of these four are Primary Replication Variants (PRVs) found in the Discovery stage (rs17235773, rs3754112, rs12093834). The fourth, rs140567712, has an observed MAF = 0.005 (MAF = 0.006 in 1kG Kenyans) and a Combined Annotation And Depletion (CADD) score of 16.7, third highest among the CD101 variants found in this study (S8 Table). Variant CADD scores are correlated with causal effects (35). Although not perfectly predictive, variants with higher CADD scores are currently believed to be more likely to be pathogenic or result in selection. (DOCX) [file ppat.1006703.s005.docx]

**S5 Fig**: **QQ-plot for individual variant p-values for *CD101* missense variants found in the Replication Stage having empirical MAF > 0.005.**
