# Supplementary material for: Whole genome sequencing of extreme phenotypes identifies variants in CD101 and UBE2V1 associated with increased risk of sexually acquired HIV-1
Source: PLoS Pathog. 2017 Nov 6;13(11):e1006703. doi: 10.1371/journal.ppat.1006703 (PMC5690691; doi:10.1371/journal.ppat.1006703)
Supplement: S7 Fig — This transformation retains the [0, 1] range of the PI for ease of coefficient/HR interpretation. The PI is the proportion of visit surveys at which only protected sex or abstinence was reported (no unprotected sex with the HIV-1 infected partner.) (DOCX) [file ppat.1006703.s007.docx]

**
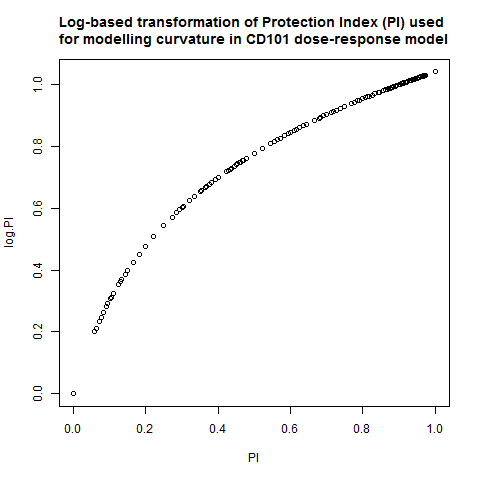
**

log(10*PI + 1) – 1

**S7 Fig: Log-type transformation of Protected-sex Index (PI)** **used to improve the dose-response model fit for the *CD101* Ig-like variant score in the Cox model.**
