# Supplementary material for: Whole genome sequencing of extreme phenotypes identifies variants in CD101 and UBE2V1 associated with increased risk of sexually acquired HIV-1
Source: PLoS Pathog. 2017 Nov 6;13(11):e1006703. doi: 10.1371/journal.ppat.1006703 (PMC5690691; doi:10.1371/journal.ppat.1006703)
Supplement: S8 Fig — The CD101 carrier group includes 58 individuals with serum cytokine measurements who have at least one alternative allele at chr1:117554421 or chr1:117560058 or chr1:117568500, which are the three variants in the Ig-like Primary Replication Variants (PRV) group that had individual FDRs < 0.05 in the replication stage. The non-carrier group (N = 105) includes individuals without alternate alleles detected at any of these three CD101 sites. P-values are for the odds of being in the fourth (highest) quartile of the cytokine distribution over both groups. The distribution of IL1R1 levels among carriers was significantly different between groups (OR = 0.19, 95% CI = [0.07, 0.54], p = 0.0017; adjusted p < 0.05)(S10 Table). (DOCX) [file ppat.1006703.s008.docx]

**
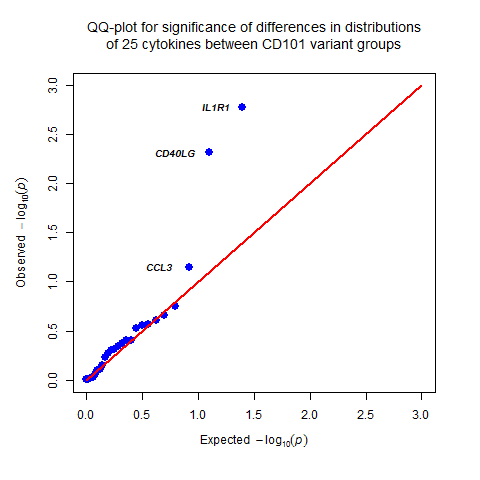
**

**S8 Fig: QQ-plot for significance of differences in cytokine distribution between *CD101* Ig-like risk variant carriers and non-carriers.**
