# Supplementary material for: Whole genome sequencing of extreme phenotypes identifies variants in CD101 and UBE2V1 associated with increased risk of sexually acquired HIV-1
Source: PLoS Pathog. 2017 Nov 6;13(11):e1006703. doi: 10.1371/journal.ppat.1006703 (PMC5690691; doi:10.1371/journal.ppat.1006703)
Supplement: S9 Fig — The distributions of 25 cytokines were screened for association with presence of any minor allele for the three most common of the five Ig-like primary replication variants (rs34999087, rs17235773, and rs12093834) among 163 individuals in the Augmented Replication sample plus Discovery sample who have cytokine measurements available. Association with IL1R1 distribution was significant after adjustment for multiple testing (OR = 0.19 for achieving the 75th percentile IL1R1 value, 95% CI = [0.07, 0.54], p = 1.7x10^-3^; adjusted p = 0.04), indicating significantly lower levels of IL1R1 among those with the Ig-like missense variants. Shown are the distributions of log(IL1R1) after adjustment for panel/batch for individuals in the cytokine analyses with and without these Ig-like primary replication missense variants. (DOCX) [file ppat.1006703.s009.docx]

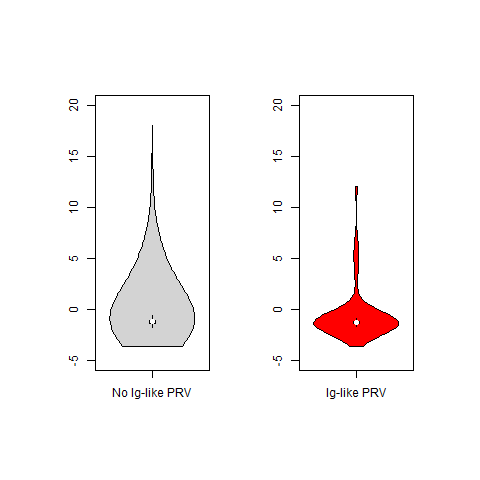


**S9 Fig. Distributions of IL1R1 among individuals with and without Ig-like variants in the cytokine analyses**
