# Supplementary material for: Whole genome sequencing of extreme phenotypes identifies variants in CD101 and UBE2V1 associated with increased risk of sexually acquired HIV-1
Source: PLoS Pathog. 2017 Nov 6;13(11):e1006703. doi: 10.1371/journal.ppat.1006703 (PMC5690691; doi:10.1371/journal.ppat.1006703)
Supplement: S10 Fig — Plots are restricted to participants from ethnicities reported by >2% of study participants. A) Parallel coordinates showing the first five scaled PCs by self-reported ethnicity. Transparent lines represent each individual. Thick lines are from smoothed lowess curves and represent the average within self-reported ethnicity groups. B) PCs 1 and 2 by self-reported ethnicity. Points from each of five countries (Kenya, Uganda, Tanzania, South Africa and Botswana) are shown in grey. C) PCs 1 and 2 by ancestry cluster determined by model based clustering of PCs 1–10. (DOCX) [file ppat.1006703.s010.docx]

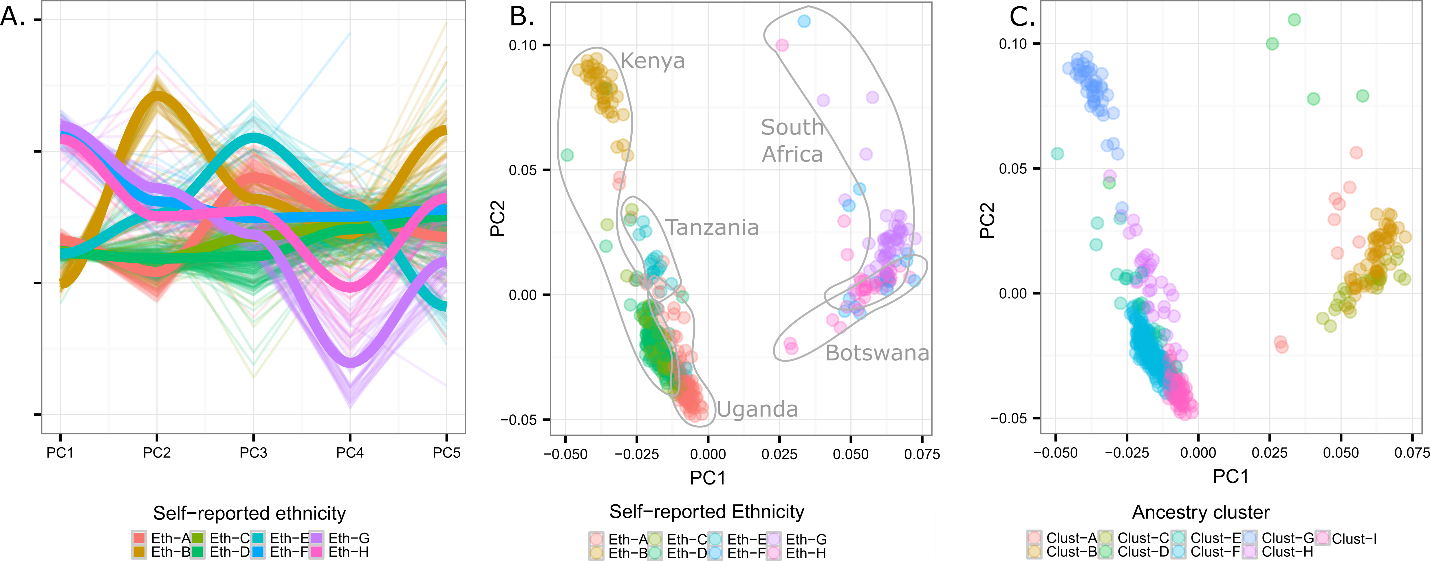


**S10 Fig: Principal component analysis (PCA) of 135,000 single nucleotide polymorphisms from a previous genome-wide association study of 798 individuals from sub-Saharan Africa, by self-reported ethnicity and model based ancestry clusters**.
