# Supplementary material for: Whole genome sequencing of extreme phenotypes identifies variants in CD101 and UBE2V1 associated with increased risk of sexually acquired HIV-1
Source: PLoS Pathog. 2017 Nov 6;13(11):e1006703. doi: 10.1371/journal.ppat.1006703 (PMC5690691; doi:10.1371/journal.ppat.1006703)
Supplement: S11 Fig — These data were used to quality control these case-control pairs for gender check, cryptic relatedness and genetic heterogeneity across multiple longitudinal whole blood DNA samples. A) Parallel coordinates showing the first five scaled principal components (PC) estimated using 384 SNPs by ancestry clusters determined using PC analysis (PC) of 133,991 SNPs. B) PCs 1 and 2 by ancestry cluster. C) Variable importance measures from Random Forest analysis of 384 SNPs to predict ancestry cluster. D) Variable importance measures from Random Forest analysis to predicted ancestry cluster using 384 SNPs, geographic region (east vs. southern Africa) and self-reported ethnicity. (DOCX) [file ppat.1006703.s011.docx]

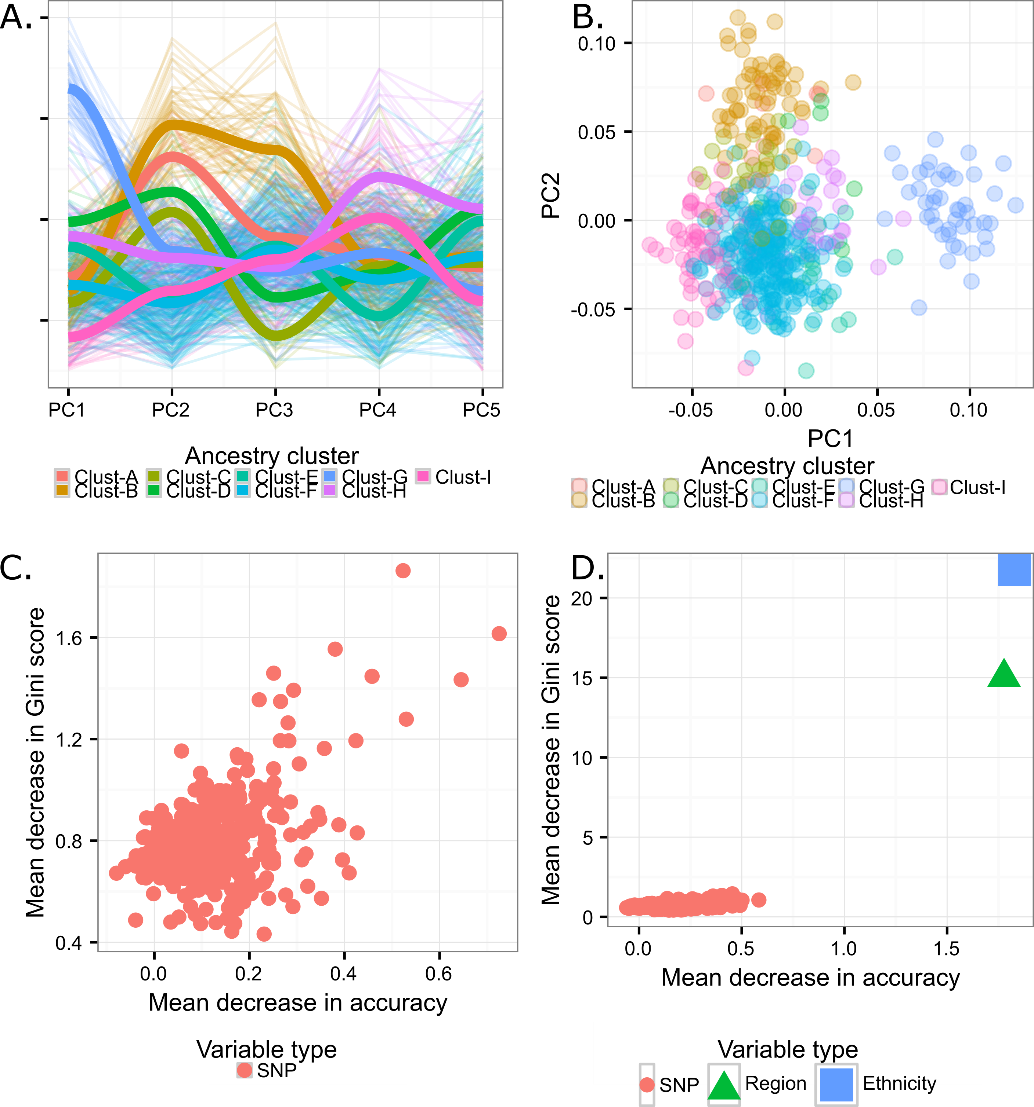


**S11 Fig:** **Analyses of 384 single nucleotide polymorphisms (SNPs) selected for custom Illumina Goldengate SNP chip**
