# Supplementary material for: Whole genome sequencing of extreme phenotypes identifies variants in CD101 and UBE2V1 associated with increased risk of sexually acquired HIV-1
Source: PLoS Pathog. 2017 Nov 6;13(11):e1006703. doi: 10.1371/journal.ppat.1006703 (PMC5690691; doi:10.1371/journal.ppat.1006703)
Supplement: S1 Table — PrEP participants were available for the Replication sample but not available for selection in the Discovery stage because the trial was still ongoing at the time of Discovery sample selection. (DOCX) [file ppat.1006703.s012.docx]

| **Study population** | **Partners in Prevention HSV/HIV Transmission Study** | **Couples Observational Study** | **Partners PrEP Study** |
| --- | --- | --- | --- |
| **Sample size (couples)** | 3408 | 485 | 4758 |
| **Location** | Botswana, Kenya, Rwanda*, South Africa, Tanzania, Uganda, Zambia* | Uganda, and South Africa | Uganda, Kenya |
| **Follow-up** | 12-24 months | 12 months | 24-36 months |
| **Study design** | Randomized trial | Observational study | Randomized trial |
| **Primary aim** | Evaluate HSV-2 suppression provided to the HIV-1 infected partner as a way of reducing HIV-1 infectiousness | Sample and data collection | Evaluate pre-exposure prophylaxis (tenofovir or tenofovir/emcitritabine versus placebo) provided to the HIV-1 uninfected partner to reduce HIV-1 transmission |
| **HIV-1 seroconversion events** | 151 | 16 | 138 |
| **Study Status** | Follow-up completed Oct 2008 | Follow-up completed Feb 2010 | Follow-up completed in 2013 |

* Samples from these sites did not contribute to this genetic analysis

**S1 Table: Cohorts providing study samples and data.**
