# Supplementary material for: Whole genome sequencing of extreme phenotypes identifies variants in CD101 and UBE2V1 associated with increased risk of sexually acquired HIV-1
Source: PLoS Pathog. 2017 Nov 6;13(11):e1006703. doi: 10.1371/journal.ppat.1006703 (PMC5690691; doi:10.1371/journal.ppat.1006703)
Supplement: S2 Table — (DOCX) [file ppat.1006703.s013.docx]

| **A. Whole genome sequence characteristics for selected highly-exposed HIV-1 exposed seronegatives (HESN), low-exposed seroconverters (SC) and all genomes (median [interquartile range])** | | | | |
| --- | --- | --- | --- | --- |
| **CHARACTERISTIC** | **HESN CONTROLS (n=50)** | | **SC CASES (n=50)** | **ALL GENOMES (n=100)** |
| **Gross mapping yield (Gb)** | 172.2 [168.7; 174.4] | | 172.3 [168.7; 175] | 172.2 (168.6,174.8) |
| **Proportion of genome with high confidence calls** | 0.966 (0.964, 0.968) | | 0.966 (0.965, 0.968) | 0.966 (0.965, 0.968) |
| **Proportion of genome with ≥40x coverage** | 0.64 [0.62; 0.67] | | 0.64 [0.62; 0.68] | 0.64 [0.62; 0.67] |
| **Proportion of genome with ≥10x coverage** | 0.98 [0.98;0.98] | | 0.98 [0.98;0.98] | 0.98 [0.98;0.98] |
| **Total variations per genome** | 5 x10^6^ [5 x10^6^; 5.1x10^6^] | | 5x10^6^ [5 x10^6^; 5.1x10^6^] | 5x10^6^ [5.x10^6^; 5.1x10^6^] |
| **Number of SNVs per genome** | 4.1x10^6^ [4.1x10^6^; 4.2x10^6^] | | 4.2x10^6^ [4.1x10^6^; 4.2 x10^6^] | 4.1x10^6^ [4.1x10^6^; 4.2x10^6^] |
| **Ti/Tv ratio*** | 2.1 [2.1; 2.1] | | 2.1 [2.1; 2.1] | 2.1 [2.1; 2.1] |
| **B. Variation characteristics across all genomes** | | | | |
| **Total VQHIGH variants** | | 31,411,871 | | |
| **SNV variants** | | 25,760,317 | | |
| **Autosomal SNVs** | | 24,806,385 | | |
| **SNPs with >=95% Call rate** | | 21,922,789 | | |
| **Autosomal, with >=95% call rate, and excluding monomorphic SNVs** | | 21,812,307 | | |
| **Total exome SNVs** | | 454,176 | | |

*Transition/transversion ratio

**S2 Table: Characteristics of discovery stage whole genome sequences (n=100).**
