# Supplementary material for: Whole genome sequencing of extreme phenotypes identifies variants in CD101 and UBE2V1 associated with increased risk of sexually acquired HIV-1
Source: PLoS Pathog. 2017 Nov 6;13(11):e1006703. doi: 10.1371/journal.ppat.1006703 (PMC5690691; doi:10.1371/journal.ppat.1006703)
Supplement: S3 Table — All variants that had no rsID in dbSNP at the time of WGA sequencing in genes moved forward for replication were Sanger sequenced from the same original DNA sample used for WGS. Given these are not previously verified sites of variation, these variants have a higher likelihood of being sequencing errors. All eight variants with the highest probability of having false-positive calls were replicated by Sanger sequencing. (DOCX) [file ppat.1006703.s014.docx]

| **Gene** | **CGI variant ID** | **Chr position** | **Minor Allele Frequency**  **(if known)** | **Variant base** | **Variant confirmed by Sanger sequencing** |
| --- | --- | --- | --- | --- | --- |
| *CD101* | 1241582 | chr1:117552814 | 1:10398 African Allele in ExAC | G | yes |
|  | 1241789 | chr1:117576709 | -- | A | yes |
|  | 1241816 | chr1:117578861 | -- | C | yes |
|  | 1241817 | chr1:117578973 | -- | A | yes |
| *UBE2V1* | 29199481 | chr20:48698572 | 0.0002 from 1000 Genomes | A | yes |
|  | 29199483 | chr20:48698737 | -- | C | yes |
|  | 29199815 | chr20:48729710 | 8:1410 in Africans Alleles ­in ExAC | A | yes |
|  | 29199850 | chr20:48732308 | -- | C | yes |

**S3 Table: Sanger validation of whole genome sequencing.**
