# Supplementary material for: Whole genome sequencing of extreme phenotypes identifies variants in CD101 and UBE2V1 associated with increased risk of sexually acquired HIV-1
Source: PLoS Pathog. 2017 Nov 6;13(11):e1006703. doi: 10.1371/journal.ppat.1006703 (PMC5690691; doi:10.1371/journal.ppat.1006703)
Supplement: S6 Table — (DOCX) [file ppat.1006703.s017.docx]

| **Characteristic** | **Replication Sample** | | **Auxillary Sample** | |
| --- | --- | --- | --- | --- |
|  | **Seroconverter (n=53)** | **HESN**  **(n=208)** | **Seroconverter (n=126)** | **HESN**  **(n=842)** |
| **Demographic and Exposure** |  |  |  |  |
| Female gender | 22 (42%) | 71 (34%) | 63 (50%) | 509 (60%) |
| Age (years) | 29.8 (25.2,37.1) | 32.9 (28,37.8) | 30.7 (25.9,36.4) | 30.4 (25.6,36.3) |
| Male circumcision | 12 (39%) | 56 (41%) | 35 (56%) | 144 (43%) |
| East African | 48 (91%) | 186 (89%) | 119 (94%) | 775 (92%) |
| Study cohort |  |  |  |  |
| Couples Observation Study | 1 (2%) | 25 (12%) | 3 (2%) | 117 (14%) |
| Partner’s in Prevention  HSV/HIV Transmission Study | 15 (28%) | 47 (23%) | 25 (20%) | 431 (51%) |
| Partners PrEP Study | 37 (70%) | 136 (65%) | 98 (78%) | 294 (35%) |
| 100% condom use reported | 0 (0%) | 0 (0%) | 83 (66%) | 498 (59%) |
| Proportion of visits with 100% condom use | 0.3 (0,0.5) | 0.4 (0.2,0.5) | 1 (0.9,1) | 1 (0.9,1) |
| Mean exposure score | 1.2 (0.3,1.7) | 1.1 (0.6,1.6) | 0.5 (0.1,1.2) | 0.9 (0.4,1.3) |
| Cumulative monthly exposure score | 6.2 (0.9,12.6) | 17.4 (8.3, 26.6) | 4.9 (0.7,11.7) | 16.2 (7.3,24.2) |
| Number of follow-up months | 6.3 (2.9,18.1) | 16.2 (11.7,23.2) | 10.8 (3.6,17.8) | 20.2 (13.6,23.3) |
| **Clinical parameters of HIV-1 infected partner** | | | | |
| Plasma HIV-1 RNA (log10 c/mL) | 4.2 (3.7,4.7) | 4.3 (3.9,4.7) | 4.5 (4.1,5) | 4.7 (4.2,5.1) |
| CD4 count, cells/mm^3^ | 463 (351, 608) | 499.5 (385, 652) | 433 (341, 569) | 427 (328, 565) |

**S6** **Table:** **Characteristics of Replication and Auxiliary Samples.**
