# Supplementary material for: Whole genome sequencing of extreme phenotypes identifies variants in CD101 and UBE2V1 associated with increased risk of sexually acquired HIV-1
Source: PLoS Pathog. 2017 Nov 6;13(11):e1006703. doi: 10.1371/journal.ppat.1006703 (PMC5690691; doi:10.1371/journal.ppat.1006703)
Supplement: S7 Table — A. CD101. B. UBE2V1. (DOCX) [file ppat.1006703.s018.docx]

**S7 Table: Primary Replication Variants found by MIPs in Replication and Augmented (Replication plus Auxiliary) Samples**

1. ***CD101***

|  | **Variant** |  | **Replication Sample** | | | | | | **Augmented Sample** | | | |
| --- | --- | --- | --- | --- | --- | --- | --- | --- | --- | --- | --- | --- |
| **HG37 position** | **rs number** | **Domain/ Group** | **HR** | **P** | **MAF** | **N Homo-zygous reference** | **N Hetero-zygous** | **N Homo-zygous alternate** | **MAF** | **N Homo-zygous reference** | **N Hetero-zygous** | **N Homo-zygous alternate** |
| 117554421 | rs3754112 | Ig-like | 3.1 | 2.33E-03^#^ | 0.038 | 240 | 20 | 0 | 0.026 | 1164 | 60 | 2 |
| 117560058 | rs17235773 | Ig-like | 3.1 | 2.11E-03^#^ | 0.043 | 237 | 20 | 1 | 0.058 | 1085 | 130 | 6 |
| 117561030 | rs116063197 | Ig-like | 0.0 | 0.996 | 0.010 | 256 | 5 | 0 | 0.012 | 1196 | 29 | 0 |
| 117568500 | rs12093834 | Ig-like | 2.0 | 0.015^#^ | 0.121 | 201 | 55 | 4 | 0.096 | 1002 | 212 | 12 |
| 117561116 | rs34882009 | Ig-like | 0.9 | 0.83 | 0.033 | 243 | 17 | 0 | 0.030 | 1152 | 71 | 1 |
| 117576550 | rs12097758 | Cyto | 3.3 | 0.097 | 0.012 | 254 | 6 | 0 | 0.012 | 1195 | 30 | 0 |
| 117576619 | rs12067543 | Cyto | 2.3 | 0.42 | 0.008 | 256 | 4 | 0 | 0.005 | 1214 | 12 | 0 |
| 117576631 | rs34248572 | Cyto | 1.4 | 0.36 | 0.077 | 220 | 40 | 0 | 0.077 | 1046 | 172 | 8 |
| 117576679 | rs150494742 | Cyto | 1.4 | 0.52 | 0.035 | 242 | 18 | 0 | 0.041 | 1127 | 98 | 1 |
| 117578861 | 1_117578861 | 3’-UTR | 0.0 | 1.00 | 0.004 | 258 | 2 | 0 | 0.003 | 1215 | 7 | 0 |
| 117579108 | rs35163967 | 3’-UTR | 0.6 | 0.35 | 0.062 | 229 | 30 | 1 | 0.052 | 1105 | 117 | 5 |
| 117556418 | rs142460852 | Splice* | 1.0 | NA | 0.000 | 239 | 0 | 0 | 0.001 | 1091 | 3 | 0 |

# By-variant FDR < 0.05
* from CGI annotation

| **Variant** | | | | | | **Replication Sample** | | | | | | | | | | | | | | | | | | **Augmented Sample** | | | | | | | | | | | | | |
| --- | --- | --- | --- | --- | --- | --- | --- | --- | --- | --- | --- | --- | --- | --- | --- | --- | --- | --- | --- | --- | --- | --- | --- | --- | --- | --- | --- | --- | --- | --- | --- | --- | --- | --- | --- | --- | --- |
| **HG37 position** | | **rs number** | | | **Domain/ Group** | **HR** | | | | | **P** | | | **MAF** | | | | **N Homo-zygous reference** | | **N Hetero-zygous** | | **N Homo-zygous alternate** | **MAF** | | | | **N Homo-zygous reference** | | | **N Hetero-zygous** | | **N Homo-zygous alternate** | | | **N with Minor Allele** | |  |
| **UBE2V1 Primary Replication Variants Found in MIPs - All Samples** | | | | | | | | | | | | | | | | | | | | | | | | | | | | | | | | | | | | | |
| 48732109 | | 6095771 | | | 5’-UTR | | | 2.15 | | | | 0.14 | | | | 0.019 | | | 250 | | 10 | 0 | | | 0.021 | | | 1177 | | | 47 | | 2 | | | 49 | |
| 48732456 | | 185632114 | | | 5’-UTR | | | NA | | | | NA | | | | 0.000 | | | 237 | | 0 | 0 | | | 0.001 | | | 1087 | | | 3 | | 0 | | | 3 | |
| 48698020 | | 115164526 | | | 3’-UTR | | | 0.80 | | | | 0.76 | | | | 0.021 | | | 249 | | 11 | 0 | | | 0.02 | | | 1174 | | | 48 | | 0 | | | 48 | |
| 48698629 | | 6095755 | | | 3’-UTR | | | 0.0 | | | | 1.0 | | | | 0.010 | | | 255 | | 5 | 0 | | | 0.01 | | | 1200 | | | 24 | | 0 | | | 24 | |
| 48697992 | | 186621934 | | | 3’-UTR | | | NA | | | | NA | | | | 0.002 | | | 238 | | 1 | 0 | | | 0.004 | | | 1082 | | | 8 | | 0 | | | 8 | |
| 48699090 | | 41283596 | | | 3’-UTR | | | NA | | | | NA | | | | 0.002 | | | 238 | | 1 | 0 | | | 0.004 | | | 1087 | | | 8 | | 0 | | | 8 | |
| 48699273 | | 187204768 | | | 3’-UTR | | | NA | | | | NA | | | | 0.000 | | | 259 | | 0 | 0 | | | 0.001 | | | 1220 | | | 3 | | 0 | | | 3 | |
| **UBE2V1 Primary Replication Variants Found in MIPs – Female Samples** | | | | | | | | | | | | | | | | | | | | | | | | | | | | | | | | | | | | | |
| 48732109 | | | 6095771 | | 5’-UTR | | 6.4 | | | 8.9E-04 | | | | | 0.022 | | | 89 | | 4 | | 0 | | 0.020 | | | 638 | | | 23 | | 2 | | 25 | | | |
| 48732456 | | | 185632114 | | 5’-UTR | | NA | | | NA | | | | | 0.000 | | | 86 | | 0 | | 0 | | 0.002 | | | 601 | | | 3 | | 0 | | 3 | | | |
| 48698020 | | | 115164526 | | 3’-UTR | | 0.8 | | | 0.86 | | | | | 0.027 | | | 88 | | 5 | | 0 | | 0.023 | | | 630 | | | 30 | | 0 | | 30 | | | |
| 48698629 | | | 6095755 | | 3’-UTR | | 0.0 | | | 1.0 | | | | | 0.022 | | | 89 | | 4 | | 0 | | 0.012 | | | 646 | | | 16 | | 0 | | 16 | | | |
| 48697992 | | | 186621934 | | 3’-UTR | | NA | | | NA | | | | | 0.006 | | | 85 | | 1 | | 0 | | 0.002 | | | 599 | | | 3 | | 0 | | 3 | | | |
| 48699090 | | | 41283596 | | 3’-UTR | | NA | | | NA | | | | | 0.006 | | | 85 | | 1 | | 0 | | 0.002 | | | 603 | | | 3 | | 0 | | 3 | | | |
| 48699273 | | | 187204768 | | 3’-UTR | | NA | | | NA | | | | | 0.000 | | | 93 | | 0 | | 0 | | 0.002 | | | 660 | | | 2 | | 0 | | 2 | | | |
| **UBE2V1 Primary Replication Variants Found in MIPs – Male Samples** | | | | | | | | | | | | | | | | | | | | | | | | | | | | | | | | | | | | | |
| 48732109 | 6095771 | | | 5’-UTR | | | | | 0.0 | | | | 1.0 | | | | 0.018 | | 161 | | 6 | 0 | | | | 0.021 | | | 539 | 24 | | 0 | | 24 | | | |
| 48732456 | 185632114 | | | 5’-UTR | | | | | NA | | | | NA | | | | 0.000 | | 151 | | 0 | 0 | | | | 0.000 | | | 486 | 0 | | 0 | | 0 | | | |
| 48698020 | 115164526 | | | 3’-UTR | | | | | 0.8 | | | | 0.86 | | | | 0.018 | | 161 | | 6 | 0 | | | | 0.016 | | | 544 | 18 | | 0 | | 18 | | | |
| 48698629 | 6095755 | | | 3’-UTR | | | | | NA | | | | NA | | | | 0.003 | | 166 | | 1 | 0 | | | | 0.007 | | | 554 | 8 | | 0 | | 8 | | | |
| 48697992 | 186621934 | | | 3’-UTR | | | | | NA | | | | NA | | | | 0.000 | | 153 | | 0 | 0 | | | | 0.005 | | | 483 | 5 | | 0 | | 5 | | | |
| 48699090 | 41283596 | | | 3’-UTR | | | | | NA | | | | NA | | | | 0.000 | | 153 | | 0 | 0 | | | | 0.005 | | | 484 | 5 | | 0 | | 5 | | | |
| 48699273 | 187204768 | | | 3’-UTR | | | | | NA | | | | NA | | | | 0.000 | | 166 | | 0 | 0 | | | | 0.001 | | | 560 | 1 | | 0 | | 1 | | | |

**S7 Table:**

**B. *UBE2V1***
