# Supplementary material for: Whole genome sequencing of extreme phenotypes identifies variants in CD101 and UBE2V1 associated with increased risk of sexually acquired HIV-1
Source: PLoS Pathog. 2017 Nov 6;13(11):e1006703. doi: 10.1371/journal.ppat.1006703 (PMC5690691; doi:10.1371/journal.ppat.1006703)
Supplement: S10 Table — This analysis included all individuals with cytokine measurements and adjusted for cytokine panel. Odds ratios (ORs) here are defined as the ratio of odds of having a cytokine value in the top quartile given that one of these three variants is present versus the odds given that all PRVs are absent. This method of testing for distributional differences was used due to large numbers of values below the limit of detection (averages are not meaningful in this situation) along with right-skewing of higher values (making a quantile approach appropriate). (DOCX) [file ppat.1006703.s021.docx]

| **Analyte** | **CD101 carriers^1^**  **(N)** | **Non-carriers^2^**  **(N)** | **Odds Ratio^3^** | **95% Lower Bound** | **95% Upper Bound** | **P-value** |
| --- | --- | --- | --- | --- | --- | --- |
| IL1R1 (IL1R | 58 | 105 | 0.19 | 0.07 | 0.54 | 0.0017* |
| sCD40L | 58 | 105 | 0.19 | 0.06 | 0.6 | 0.0049 |
| CCL3 | 58 | 105 | 0.46 | 0.2 | 1.07 | 0.0707 |
| VEGF | 58 | 105 | 1.75 | 0.78 | 3.92 | 0.177 |
| IL-7 | 58 | 105 | 0.51 | 0.17 | 1.5 | 0.219 |
| IFN-γ | 58 | 105 | 1.74 | 0.68 | 4.42 | 0.2478 |
| IL-2 | 58 | 105 | 1.68 | 0.67 | 4.22 | 0.271 |
| TNF-α | 58 | 105 | 1.66 | 0.66 | 4.19 | 0.2799 |
| IL-6 | 58 | 105 | 1.57 | 0.67 | 3.65 | 0.2994 |
| IL-8 | 58 | 105 | 0.69 | 0.3 | 1.6 | 0.3905 |
| IL-17 | 58 | 105 | 1.44 | 0.62 | 3.36 | 0.3956 |
| TGF-α | 58 | 105 | 1.36 | 0.64 | 2.91 | 0.4253 |
| CX3CL1 | 58 | 105 | 1.39 | 0.59 | 3.27 | 0.4501 |
| CCL2 | 58 | 105 | 1.37 | 0.57 | 3.32 | 0.4854 |
| CCL5 | 58 | 105 | 0.74 | 0.31 | 1.78 | 0.4977 |
| GM-CSF | 58 | 105 | 1.31 | 0.56 | 3.09 | 0.5359 |
| IL-1b | 58 | 105 | 0.76 | 0.28 | 2.04 | 0.5824 |
| IL-12p40 | 58 | 105 | 0.82 | 0.31 | 2.21 | 0.7006 |
| IL-10 | 58 | 105 | 0.89 | 0.38 | 2.06 | 0.7839 |
| G-CSF | 58 | 105 | 1.12 | 0.49 | 2.54 | 0.7915 |
| CCL4 | 58 | 105 | 1.07 | 0.49 | 2.34 | 0.8663 |
| EGF | 58 | 105 | 0.97 | 0.44 | 2.13 | 0.9394 |
| CCL11 | 58 | 105 | 1.03 | 0.44 | 2.39 | 0.9469 |
| CXCL10 | 58 | 105 | 0.98 | 0.3 | 3.23 | 0.9693 |
| IL-12p70 | 58 | 105 | 0.99 | 0.39 | 2.49 | 0.9812 |

*p<0.05 after adjustment for multiple testing

**S10 Table: Cytokine distributions comparing individuals with any of the three Ig-like *CD101* variants (rs17235773, rs3754112, rs12093834) to individuals without any of *CD101* primary replication variants (PRVs).**
