# Supplementary material for: Whole genome sequencing of extreme phenotypes identifies variants in CD101 and UBE2V1 associated with increased risk of sexually acquired HIV-1
Source: PLoS Pathog. 2017 Nov 6;13(11):e1006703. doi: 10.1371/journal.ppat.1006703 (PMC5690691; doi:10.1371/journal.ppat.1006703)
Supplement: S11 Table — Mean log10 plasma HIV-1 RNA for N = 158 individuals with versus without primary replication variants (PRVs) in CD101 or UBE2V1 and who HIV-1 seroconverted during study follow-up. The P-value estimate for this comparison, as well as the lower and upper bounds on the 95% confidence interval, are also shown. (DOCX) [file ppat.1006703.s022.docx]

| **Seroconverters with set point data**  **(N=158)** | **Mean plasma HIV-1 RNA (log_10_)** | | **P-value* for difference** | **LB for difference** | **UB for difference** |
| --- | --- | --- | --- | --- | --- |
|  | **No Primary Replication Variant** | **Primary Replication Variant carriers** |  |  |  |
| ***CD101*** | 4.12 | 4.11 | 0.96 | -0.6 | 0.63 |
| ***UBE2V1*** | 3.96 | 4.15 | 0.70 | -1.78 | 1.40 |

* Adjusted for sex and plasma HIV-1 RNA of transmitting partner

LB = lower bound for 95% confidence interval; UB = upper bound for 95% confidence interval.

**S11 Table: Comparison of plasma HIV-1 RNA set point of seroconverters with and without PRVs in *CD101* or *UBE2V1* (N=158)**
