# Supplementary material for: Whole genome sequencing of extreme phenotypes identifies variants in CD101 and UBE2V1 associated with increased risk of sexually acquired HIV-1
Source: PLoS Pathog. 2017 Nov 6;13(11):e1006703. doi: 10.1371/journal.ppat.1006703 (PMC5690691; doi:10.1371/journal.ppat.1006703)
Supplement: S12 Table — (DOCX) [file ppat.1006703.s023.docx]

| **MIP Name** | **MIP Sequence** |
| --- | --- |
| CD101_UTR_5'_Ex1_MIP_1 | catctttctttctccttctgagCTTCAGCTTCCCGATATCCGACGGTAGTGTaccacacttttgcttcct |
| CD101_Ex1_MIP_2 | cacatatgagatgcctgccaCTTCAGCTTCCCGATATCCGACGGTAGTGTtatatggcatagttgttcct |
| CD101_Ex2_MIP_3 | cagggaccttctgagcagcatCTTCAGCTTCCCGATATCCGACGGTAGTGTttattcccctttctttctc |
| CD101_Ex2_MIP_4 | gcagccaatgctgactgggtaCTTCAGCTTCCCGATATCCGACGGTAGTGTtgcgtatatactgcgtaag |
| CD101_Ex2_MIP_5 | agtatgagtgtcacacaccaaaCTTCAGCTTCCCGATATCCGACGGTAGTGTaccaaggatgctgccttc |
| CD101_Ex2_MIP_6 | ccagcatccttcatctggagtttCTTCAGCTTCCCGATATCCGACGGTAGTGTggtggagactctggctt |
| CD101_Ex3_MIP_7 | gcccaacatactcacctctcCTTCAGCTTCCCGATATCCGACGGTAGTGTgccagtcacattattatcat |
| CD101_Ex3_MIP_8 | atgcctcacaggtgagggctaaCTTCAGCTTCCCGATATCCGACGGTAGTGTgtatacaagggcccaggg |
| CD101_Ex3_MIP_9 | ccagtcctcagatcagggtcCTTCAGCTTCCCGATATCCGACGGTAGTGTcactgagattatttctctct |
| CD101_Ex3_MIP_10 | gcctgaatgtagtgggtcccagCTTCAGCTTCCCGATATCCGACGGTAGTGTatcctcagagtggtttga |
| CD101_Ex3_MIP_11 | gttctaatcttttgttggctctCTTCAGCTTCCCGATATCCGACGGTAGTGTtgttcatcaccaaaaagc |
| CD101_Ex4_MIP_12 | cttcaaggcatttggttcttcaaCTTCAGCTTCCCGATATCCGACGGTAGTGTaaatacctgccttgctt |
| CD101_Ex4_MIP_13 | ggtcacggccactgcttacaaCTTCAGCTTCCCGATATCCGACGGTAGTGTgcctaactttgaaacctgg |
| CD101_Ex4_MIP_14 | atgaaaacacgcacaggttcCTTCAGCTTCCCGATATCCGACGGTAGTGTaatgactacaaagagagagc |
| CD101_Ex4_MIP_15 | acctctgctaccacacatctgtaCTTCAGCTTCCCGATATCCGACGGTAGTGTgcatctgaaagcagccc |
| CD101_Ex5_MIP_16 | ccctgtctgtgagctggtggCTTCAGCTTCCCGATATCCGACGGTAGTGTaaacagttttgttcttctat |
| CD101_Ex5_MIP_17 | agtgtctctccttcccacacaacCTTCAGCTTCCCGATATCCGACGGTAGTGTgcacccagctgcacaat |
| CD101_Ex5_MIP_18 | agacagtggcacatatgagtgcaCTTCAGCTTCCCGATATCCGACGGTAGTGTtggcatggggcaggatg |
| CD101_Ex5_MIP_19 | gatggcagtgaaggtgatctcCTTCAGCTTCCCGATATCCGACGGTAGTGTgcagaaaaggacttccttt |
| CD101_Ex6_MIP_21 | cctgtcctgtgtcgtgagggCTTCAGCTTCCCGATATCCGACGGTAGTGTaaaacttgaaagtgctatat |
| CD101_Ex6_MIP_22 | agactaacttgtaaacttgactcCTTCAGCTTCCCGATATCCGACGGTAGTGTatgtgagagctagctgg |
| CD101_Ex6_MIP_23 | aaagtgtcgcagtctttatttcCTTCAGCTTCCCGATATCCGACGGTAGTGTcaaggtgccactcactgt |
| CD101_Ex6_MIP_24 | caatagtgccattgtgggtgCTTCAGCTTCCCGATATCCGACGGTAGTGTcataaacttctacttcacac |
| CD101_Ex6_MIP_25 | gtgacttgaaattaatccctgttCTTCAGCTTCCCGATATCCGACGGTAGTGTaggaagagacaggagtg |
| CD101_Ex6_MIP_26 | ggtaaagtgacggctatcctCTTCAGCTTCCCGATATCCGACGGTAGTGTtgtggtgcttacttagatag |
| CD101_Ex7_MIP_27 | ggaaaccttcagttagccattCTTCAGCTTCCCGATATCCGACGGTAGTGTcatagcaaagtaactgttt |
| CD101_Ex7_MIP_28 | ggaccgggacaagatgctacaCTTCAGCTTCCCGATATCCGACGGTAGTGTctctgtggggtgtgaaact |
| CD101_Ex7_MIP_29 | actgtgctgtggaggaatggCTTCAGCTTCCCGATATCCGACGGTAGTGTccaatgttataaaaactggg |
| CD101_Ex7_MIP_30 | atatttgccccgatcgctgtcCTTCAGCTTCCCGATATCCGACGGTAGTGTgacattgtgaggatacact |
| CD101_Ex8_MIP_31 | actctgtactctgtgatgtggCTTCAGCTTCCCGATATCCGACGGTAGTGTtctctaatttactgccatt |
| CD101_Ex8_MIP_32 | gatggccacttctctgtgctcaCTTCAGCTTCCCGATATCCGACGGTAGTGTcttccccatactccagca |
| CD101_Ex8_MIP_33 | actggtgtagggtggcagagtgCTTCAGCTTCCCGATATCCGACGGTAGTGTcacttgcaacatgatggc |
| CD101_Ex8_MIP_34 | cattcctgcatcctccatctccCTTCAGCTTCCCGATATCCGACGGTAGTGTcgcggtagataaacccct |
| CD101_Ex8_MIP_35 | caattttcaggttccctaaagaagCTTCAGCTTCCCGATATCCGACGGTAGTGTtgctgccttcaggtaa |
| CD101_Ex9_MIP_36 | gcttatactggaaggccaggaaCTTCAGCTTCCCGATATCCGACGGTAGTGTtactctcttgtttctccc |
| CD101_Ex9_MIP_37 | gaggagggagatgagcagaaggCTTCAGCTTCCCGATATCCGACGGTAGTGTcatcttcctcctcgtctt |
| CD101_Ex9_UTR_3'_MIP_38 | gttctgtcctcaactatgtctgCTTCAGCTTCCCGATATCCGACGGTAGTGTaggtgtgaccacaaatag |
| CD101_UTR_3'_MIP_39 | acctggaaccagctcctgacaCTTCAGCTTCCCGATATCCGACGGTAGTGTatgtaagcagacagggagg |
| CD101_UTR_3'_MIP_40 | ccacacggggcttgggaagaCTTCAGCTTCCCGATATCCGACGGTAGTGTttacggtcatgaagctatag |
| CD101_UTR_3'_MIP_41 | ccctctccatcaccccataaaaCTTCAGCTTCCCGATATCCGACGGTAGTGTatcctgaagtcactaccc |
| CD101_UTR_3'_MIP_42 | agagagtgcattggtgcatgtCTTCAGCTTCCCGATATCCGACGGTAGTGTatgagttttgatgaaaagg |
| CD101_UTR_3'_MIP_43 | atatgaaacggttccggccagCTTCAGCTTCCCGATATCCGACGGTAGTGTctctccttgcttgtgacaa |
| CD101_UTR_3'_MIP_44 | atatgaaactgttccggccagCTTCAGCTTCCCGATATCCGACGGTAGTGTctctccttgcttgtgacaa |
| UBE2V1_UTR_3'_MIP_127 | ggctgcttatggaatactgttCTTCAGCTTCCCGATATCCGACGGTAGTGTaatcccaaagcatctaaaa |
| UBE2V1_UTR_3'_MIP_128 | gcctgattttaatataataaaaCTTCAGCTTCCCGATATCCGACGGTAGTGTctgaaaagcaattgatgt |
| UBE2V1_UTR_3'_MIP_129 | gcttttatttcggtatgcatccacCTTCAGCTTCCCGATATCCGACGGTAGTGTttccccccagaaaaag |
| UBE2V1_UTR_3'_MIP_130 | gtgtaaaacccccttttgaagcaCTTCAGCTTCCCGATATCCGACGGTAGTGTtcctggcaaattgctgc |
| UBE2V1_UTR_3'_MIP_131 | gtgtaaaaccaccttttgaagcaCTTCAGCTTCCCGATATCCGACGGTAGTGTtcctggcaaattgctgc |
| UBE2V1_UTR_3'_MIP_132 | caaggcatgcaccggattcaaCTTCAGCTTCCCGATATCCGACGGTAGTGTtgaacagcaagtggaaaga |
| UBE2V1_UTR_3'_MIP_133 | atcctcagcagggattaagaaCTTCAGCTTCCCGATATCCGACGGTAGTGTgttttaaacagggctttca |
| UBE2V1_UTR_3'_MIP_134 | gtctttctccaagttcaaccacCTTCAGCTTCCCGATATCCGACGGTAGTGTcccaatggattgcaagga |
| UBE2V1_UTR_3'_MIP_135 | gcatgaagatacttgtaagcacaCTTCAGCTTCCCGATATCCGACGGTAGTGTaccctctgctgtcatca |
| UBE2V1_UTR_3'_MIP_136 | gctctttccaaccttacctgaCTTCAGCTTCCCGATATCCGACGGTAGTGTaaacctgagctaaaaacaa |
| UBE2V1_UTR_3'_MIP_137 | gacctcccttgtttagttttggCTTCAGCTTCCCGATATCCGACGGTAGTGTcctttatcgaggggtgct |
| UBE2V1_UTR_3'_MIP_138 | atctatgcttcggccaagggcCTTCAGCTTCCCGATATCCGACGGTAGTGTggtgcaaaaaggaacttga |
| UBE2V1_UTR_3'_MIP_139 | gtaacccctccactcccctctgCTTCAGCTTCCCGATATCCGACGGTAGTGTggactactgctaggtgtg |
| UBE2V1_UTR_3'_MIP_140 | gtaacccctccactcacctctgCTTCAGCTTCCCGATATCCGACGGTAGTGTggactactgctaggtgtg |
| UBE2V1_UTR_3'_MIP_141 | ggtgggaggcgggagggtaaCTTCAGCTTCCCGATATCCGACGGTAGTGTtaagacttaaaggaaccagg |
| UBE2V1_UTR_3'_MIP_142 | ggtgggaggcgggagggtaaCTTCAGCTTCCCGATATCCGACGGTAGTGTtaagacttaaagaaaccagg |
| UBE2V1_UTR_3'_MIP_143 | ccctgcaacaacagccctctCTTCAGCTTCCCGATATCCGACGGTAGTGTaattgattcccatcataact |
| UBE2V1_UTR_3'_MIP_144 | aaatggacaaaaaattagtatCTTCAGCTTCCCGATATCCGACGGTAGTGTtgagttagatgtgccccac |
| UBE2V1_UTR_3'_MIP_145 | gtcaagtgtaaccactgtccaCTTCAGCTTCCCGATATCCGACGGTAGTGTatacgtcttgtagacctca |
| UBE2V1_UTR_3'_MIP_146 | attaattgctgtaacactgtcctCTTCAGCTTCCCGATATCCGACGGTAGTGTtgaatgggagcttcctt |
| UBE2V1_Ex4_MIP_147 | aaaaagaaaaaccacaggcccCTTCAGCTTCCCGATATCCGACGGTAGTGTtcagtgctagcaaaatggc |
| UBE2V1_Ex4_MIP_148 | gctaaggtgcaaactggagtCTTCAGCTTCCCGATATCCGACGGTAGTGTgacaactttgatgctatatg |
| UBE2V1_Ex3_MIP_149 | gctgtatattcggttttcataaaCTTCAGCTTCCCGATATCCGACGGTAGTGTggaacaagtgggacaga |
| UBE2V1_Ex3_MIP_150 | gtggacctaaatacccagaagcCTTCAGCTTCCCGATATCCGACGGTAGTGTacagtcagggataaatgg |
| UBE2V1_Ex2_MIP_151 | catctcctactcctttctggcCTTCAGCTTCCCGATATCCGACGGTAGTGTggcacagtgaatttaagac |
| UBE2V1_Ex2_MIP_152 | cacagttagctggggtctagaCTTCAGCTTCCCGATATCCGACGGTAGTGTtctaggcttacaagttccc |
| UBE2V1_Ex1_MIP_153 | cccttctatgaggatagtagtatCTTCAGCTTCCCGATATCCGACGGTAGTGTggaagcatttgggttcc |
| UBE2V1_Ex1_UTR_5'_MIP_154 | caggagaggttcaagcgtctCTTCAGCTTCCCGATATCCGACGGTAGTGTgcttggtaattatgagctct |
| UBE2V1_UTR_5'_MIP_155 | actcagagctaacaggtggagaCTTCAGCTTCCCGATATCCGACGGTAGTGTggcacagggaagaggaag |
| UBE2V1_UTR_5'_MIP_156 | acaggagttgctgcatcgagtCTTCAGCTTCCCGATATCCGACGGTAGTGTcaagagccaaggaatccat |
| UBE2V1_UTR_5'_MIP_157 | ggttgttgtagtctagcgttggCTTCAGCTTCCCGATATCCGACGGTAGTGTcccgtaaatgtttactcg |

**S12 Table: MIPS Primers**
