# Supplementary material for: Whole genome sequencing of extreme phenotypes identifies variants in CD101 and UBE2V1 associated with increased risk of sexually acquired HIV-1
Source: PLoS Pathog. 2017 Nov 6;13(11):e1006703. doi: 10.1371/journal.ppat.1006703 (PMC5690691; doi:10.1371/journal.ppat.1006703)
Supplement: S1 References — (DOCX) [file ppat.1006703.s024.docx]

**SUPPLEMENTARY REFERENCES**

**S1.** Genomes Project Consortium, Abecasis GR, Auton A, Brooks LD, DePristo MA, Durbin RM, et al. An integrated map of genetic variation from 1,092 human genomes. Nature. 2012;491(7422):56-65. doi: 10.1038/nature11632. PubMed PMID: 23128226; PubMed Central PMCID: PMC3498066.

**S2.** Cooper GM, Stone EA, Asimenos G, Program NCS, Green ED, Batzoglou S, et al. Distribution and intensity of constraint in mammalian genomic sequence. Genome Res. 2005;15(7):901-13. doi: 10.1101/gr.3577405. PubMed PMID: 15965027; PubMed Central PMCID: PMC1172034.

**S3.** Adzhubei IA, Schmidt S, Peshkin L, Ramensky VE, Gerasimova A, Bork P, et al. A method and server for predicting damaging missense mutations. Nat Methods. 2010;7(4):248-9. doi: 10.1038/nmeth0410-248. PubMed PMID: 20354512; PubMed Central PMCID: PMC2855889.

**S4.** Grantham R. Amino acid difference formula to help explain protein evolution. Science. 1974;185(4154):862-4. PubMed PMID: 4843792.

**S5.** Kircher M, Witten DM, Jain P, O'Roak BJ, Cooper GM, Shendure J. A general framework for estimating the relative pathogenicity of human genetic variants. Nat Genet. 2014;46(3):310-5. doi: 10.1038/ng.2892. PubMed PMID: 24487276; PubMed Central PMCID: PMC3992975.
