# Supplementary figures and images for: Correction: Whole genome sequencing of extreme phenotypes identifies variants in CD101 and UBE2V1 associated with increased risk of sexually acquired HIV-1
Source: PLoS Pathog. 2019 Feb 11;15(2):e1007588. doi: 10.1371/journal.ppat.1007588 (PMC6370236; doi:10.1371/journal.ppat.1007588)

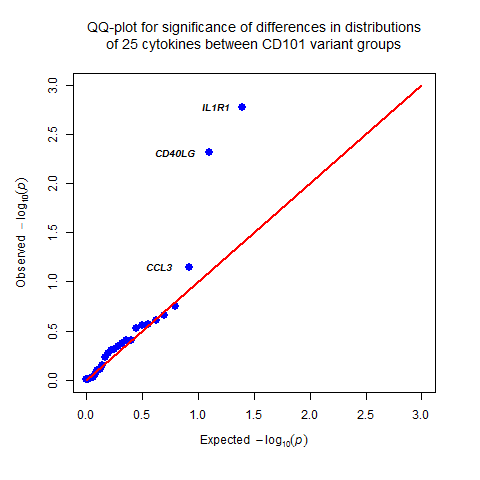

Supplement: S8 Fig — The CD101 carrier group includes 58 individuals with serum cytokine measurements who have at least one alternative allele at chr1:117554421 or chr1:117560058 or chr1:117568500, which are the three variants in the Ig-like Primary Replication Variants (PRV) group that had individual FDRs < 0.05 in the replication stage. The non-carrier group (N = 105) includes individuals without alternate alleles detected at any of these three CD101 sites. P-values are for the odds of being in the fourth (highest) quartile of the cytokine distribution over both groups. The distribution of IL1RN levels among carriers was significantly different between groups (OR = 0.19, 95% CI = [0.07, 0.54], p = 0.0017; adjusted p < 0.05) (PNG) [file ppat.1007588.s001.png]

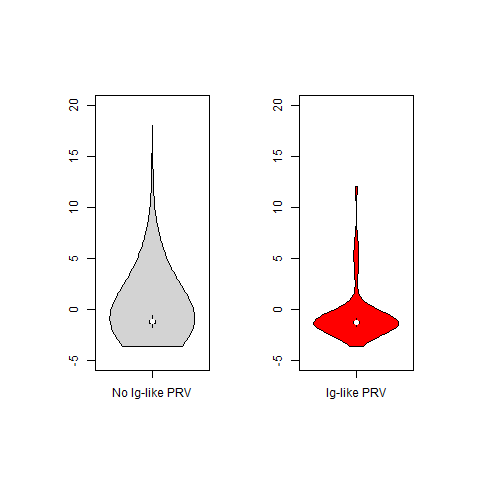

Supplement: S9 Fig — The distributions of 25 cytokines were screened for association with presence of any minor allele for the three most common of the five Ig-like primary replication variants (rs34999087, rs17235773, and rs12093834) among 163 individuals in the Augmented Replication sample plus Discovery sample who have cytokine measurements available. Association with IL1RN distribution was significant after adjustment for multiple testing (OR = 0.19 for achieving the 75th percentile IL1RN value, 95% CI = [0.07, 0.54], p = 1.7x10^-3^; adjusted p = 0.04), indicating significantly lower levels of IL1RN among those with the Ig-like missense variants. Shown are the distributions of log(IL1RN) after adjustment for panel/batch for individuals in the cytokine analyses with and without these Ig-like primary replication missense variants. (PNG) [file ppat.1007588.s002.png]
